# Extensive Genome-Wide Phylogenetic Discordance Is Due to Incomplete Lineage Sorting and Not Ongoing Introgression in a Rapidly Radiated Bryophyte Genus

**DOI:** 10.1093/molbev/msab063

**Published:** 2021-03-03

**Authors:** Olena Meleshko, Michael D. Martin, Thorfinn Sand Korneliussen, Christian Schröck, Paul Lamkowski, Jeremy Schmutz, Adam Healey, Bryan T. Piatkowski, A. Jonathan Shaw, David J. Weston, Kjell Ivar Flatberg, Péter Szövényi, Kristian Hassel, Hans K. Stenøien

**Affiliations:** 1 Department of Natural History, NTNU University Museum, Norwegian University of Science and Technology, Trondheim, Norway; 2 Centre for GeoGenetics, Natural History Museum of Denmark, Copenhagen, Denmark; 3 Biology Centre of the Upper Austrian State Museum, Linz, Austria; 4 Institute of Botany and Landscape Ecology, University of Greifswald, Greifswald, Germany; 5 United States Department of Energy, Joint Genome Institute, Berkeley, CA, USA; 6 HudsonAlpha Institute for Biotechnology, Huntsville, AL, USA; 7 Department of Biology, Duke University, Durham, NC, USA; 8 Biosciences Division, Oak Ridge National Laboratory, Oak Ridge, TN, USA; 9 Climate Change Science Institute, Oak Ridge National Laboratory, Oak Ridge, TN, USA; 10 Department of Systematic and Evolutionary Botany & Zurich-Basel Plant Science Center, University of Zurich, Zurich, Switzerland

**Keywords:** phylogenomics, introgression, incomplete lineage sorting, rapid diversification, speciation, peatmoss

## Abstract

The relative importance of introgression for diversification has long been a highly disputed topic in speciation research and remains an open question despite the great attention it has received over the past decade. Gene flow leaves traces in the genome similar to those created by incomplete lineage sorting (ILS), and identification and quantification of gene flow in the presence of ILS is challenging and requires knowledge about the true phylogenetic relationship among the species. We use whole nuclear, plastid, and organellar genomes from 12 species in the rapidly radiated, ecologically diverse, actively hybridizing genus of peatmoss (*Sphagnum*) to reconstruct the species phylogeny and quantify introgression using a suite of phylogenomic methods. We found extensive phylogenetic discordance among nuclear and organellar phylogenies, as well as across the nuclear genome and the nodes in the species tree, best explained by extensive ILS following the rapid radiation of the genus rather than by postspeciation introgression. Our analyses support the idea of ancient introgression among the ancestral lineages followed by ILS, whereas recent gene flow among the species is highly restricted despite widespread interspecific hybridization known in the group. Our results contribute to phylogenomic understanding of how speciation proceeds in rapidly radiated, actively hybridizing species groups, and demonstrate that employing a combination of diverse phylogenomic methods can facilitate untangling complex phylogenetic patterns created by ILS and introgression.

## Introduction

After a long history of rejecting the plausibility of speciation with gene flow in sympatry, it is now generally accepted that speciation does occur without complete geographical and reproductive isolation (Morjan and Rieseberg 2004; Sousa and Hey 2013; Ravinet et al. 2017). Indeed, mounting evidence for postspeciation introgression between closely related species has shifted the discussion toward a debate of the relative importance of gene flow for speciation. How important is gene flow per se in speciation, in comparison with other evolutionary forces? Case studies show that gene exchange between closely related species can trigger adaptive radiation (Dasmahapatra et al. 2012; Fontaine et al. 2015), whereas selective processes are more important for the divergence of lineages into separate species (Ma et al. 2018). As the magnitude of gene flow changes over the course of speciation-with-gene-flow (Feder et al. 2012), the relative role of introgression might depend on the stage of speciation.

It is well known that identifying the genomic footprints of gene flow is difficult in the presence of incomplete lineage sorting (ILS) among the diversifying species (Pinho and Hey 2010; Ravinet et al. 2017). ILS describes the pattern in which lineages fail to coalesce during speciation events due to stochasticity of the coalescent process (Degnan and Rosenberg 2009; Zwickl et al. 2014). Therefore, ILS represents the retention of ancestral polymorphism, which may become fixed in the descendant lineages after speciation events due to stochastic genetic drift (Suh et al. 2015). Similarly, to gene flow, ILS obstructs reconstruction of true evolutionary history and generates discordant phylogenetic signals among loci across the genome (Pollard et al. 2006; Avise and Robinson 2008; Suh et al. 2015; Pease et al. 2016). Several methods have been developed to differentiate between the two (Green et al. 2010; Durand et al. 2011; Edelman et al. 2019), but they require knowledge of the correct branching order among the species (Fontaine et al. 2015; Edelman et al. 2019). Despite recent advances in relevant analytical methods, the reconstruction of species relationships in the presence of gene flow and/or ILS remains challenging (Fontaine et al. 2015; Li et al. 2019), which in turn makes it difficult to quantify the level of introgression among them. However, the problem can be approached using independent phylogenomic methods on numerous genetic markers in hybridizing species across a speciation continuum, which has been accomplished in several organism groups (Pease et al. 2016; Irisarri et al. 2018). Comparing phylogenetic signals among genetic markers with differential inheritance (Zhou et al. 2017; Westbury et al. 2020) or in windows across the genome (Copetti et al. 2017; Edelman et al. 2019; Stankowski et al. 2019) can also further the inference and its interpretation. In addition, the distribution of phylogenetic discordance in the genome can inform about different selection processes in the ancestral population (Pollard et al. 2006; Slatkin and Pollack 2006; Hobolth et al. 2011; Wang and Hahn 2018).

Peatmoss (*Sphagnum* L.), a species-rich genus of nonvascular haploid plants, offers a potential for studying the relative importance of introgression and ILS in diversification. Peatmosses typically grow in peatlands, where they serve as ecosystem engineers (van Breemen 1995) and sequester carbon, thereby making peatlands the largest terrestrial carbon sink (Yu et al. 2010). Numerous peatmoss species normally disperse over wide geographical distributions (Szövényi et al. 2008; Sundberg 2013; Mikulášková et al. 2015; Kyrkjeeide et al. 2016) across which they co-occur and often hybridize. At least 20% of all species potentially engage in interspecific admixture or allopolyploid hybridization (for a review, see Meleshko et al. 2018). Difficulties in species delimitation and phylogenetic reconstruction in this genus are often attributed to interspecific introgression (Ricca et al. 2011; Shaw et al. 2012; Karlin et al. 2014). However, genetic studies on peatmosses have been performed mostly using a small number of genetic markers and focusing on phylogeography, species delimitation, or allopolyploid speciation rather than mere interspecific introgression. Hybridization does not seem to be constrained by phylogenetic relatedness in *Sphagnum*, and is common even between distantly related species from different subgenera (Meleshko et al. 2018). Yet, the extent of how often it translates into introgression is unknown. In many eukaryotes, introgression occurs more readily in genomic regions with high recombination rate (Begun and Aquadro 1992; Schumer et al. 2018). Recombination rates are negatively correlated to genome size in eukaryotes (Lynch 2006; Tiley et al. 2015), thus the relatively small peatmoss genomes might exhibit high recombination rates that could facilitate interspecific introgression.

Several features make peatmoss an excellent model for studying the long-standing question of evolutionary implications of ILS and introgression. Northern Hemisphere peatmoss species exhibit vast variability and plasticity in morphology, ecology, and life history (Stenøien et al. 2014; Johnson et al. 2015). This remarkable species diversity and variability originated rapidly and relatively recently (7–20 Ma, Shaw, Devos, et al. 2010). Typically, rampant radiations are accompanied by ILS (Whitfield and Lockhart 2007), especially if the effective population size of the ancestral population is large (Slatkin and Pollack 2006), leading to differential retention of polymorphisms that were present in the ancestral population (Pollard et al. 2006). Considering the large effective population sizes of peatmoss species, the effect of ILS might be exacerbated in the group (Stenøien and Såstad 1999). Indeed, in addition to gene flow, the retention of shared ancestral polymorphism due to ILS has been invoked to explain the low levels of among-population divergence across wide geographical distributions observed in several peatmoss species (Stenøien and Såstad 1999; Szövényi et al. 2008; Stenøien et al. 2011). Finally, it has been hypothesized that species diversity in peatmosses originated through adaptation to diverse habitats facilitated by gene flow (Yousefi et al. 2017) and differential paralog retention after the last whole-genome duplication event preceding the radiation of the group (Devos et al. 2016). Gene duplication and loss often lead to intensified genome-wide phylogenetic discordance and ILS (Rasmussen and Kellis 2012).

Here, we explore the genome-wide patterns of phylogenetic discordance in relation to gene flow and ILS in several more or less closely related peatmoss species pairs at different geographical scales. Specifically, we produced low-depth whole-genome shotgun sequencing data for 12 widely distributed, co-occurring, haploid peatmoss species representing different subgenera within the genus. We use these data to: 1) reconstruct phylogenetic relationships in the group based on genomic markers, 2) quantify levels of interspecific introgression, 3) identify signatures of ILS among the species, and 4) estimate genome-wide variation in phylogenetic discordance. Our analyses show that levels of postspeciation gene flow were surprisingly low and that ILS has mainly been responsible for shaping the genomic landscape of diversification in this diverse group of plants.

## Results

### Sequencing Summary, Mapping, SNP Calling, and Filtering

**Fig. 1.**
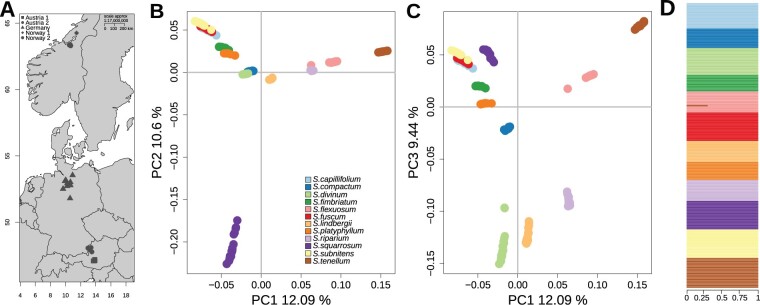
Sampling locations for and genetic differentiation among the individuals investigated. (*A*) Sampling locations. The symbols represent sampling locations for all 12 species, most collected from two parapatric populations in Austria (Tamsweg district [Austria 1] and Upper Austria [Austria 2]) and Norway (Namsos area [Norway 1] and Trondheim [Norway 2]), as well as from two populations in Germany (supplementary table S1, Supplementary Material online). (*B*) PCA of all individuals in the space of the first two principal components and (*C*) in the space of the first and the third principal components. All principal components were statistically significant (*P* < 0.001, supplementary fig. S1, Supplementary Material online). (*D*) Individual assignment for each of the 190 individuals to well-defined species for the most-supported model with *K *=* *12 genetic clusters inferred with our ADMIXTURE analysis.

We generated whole-genome shotgun sequencing data for 12 peatmoss species representing all five subgenera within *Sphagnum*, as well as an outgroup non-*Sphagnum* peatmoss species (Devos et al. 2016), *Flatbergium sericeum* (Müll. Hal.) A.J. Shaw (table 1). For each of the 12 species, we collected one to four individuals from at least two geographically separated populations (fig. 1*A* andtable 1). In total, we performed shotgun whole-genome sequencing on 191 individuals (supplementary table S1, Supplementary Material online). The sequencing reads were mapped to the *S. angustifolium* (formerly *fallax*) draft reference genome (v0.5, DOE-JGI, http://phytozome.jgi.doe.gov/, last accessed March 1, 2021). After quality filtering of the raw sequencing reads, we retained 65 ± 45 M (SD) reads per sample, of which 16 ± 0.09% (SD) were PCR duplicates, 38 ± 16% (SD) mapped uniquely to the *S. angustifolium* nuclear reference genome, and 1% and 2% mapped uniquely to the *S. fallax* mitochondrial and chloroplast genomes, respectively. Mean sequencing coverage varied from 1.6 to 14.36 (6.25 ± 2.6 SD) for the nuclear genome, whereas the mitochondrial and the chloroplast genome exhibited a sequencing coverage of 300 ± 170 SD and 830 ± 530 SD, respectively (supplementary table S2, Supplementary Material online). We did not observe any substantial difference in mapping rates among the species from different subgenera (supplementary table S1, Supplementary Material online).

**Table 1.** Sampling Summary.

Species	Subgenus	Sample Size, Norway	Sample Size, Austria	Sample Size, Germany	Total Number of Samples
*Sphagnum capillifolium*	*Acutifolia*	8	7	2	17
*S. compactum*	*Rigida*	8	3	2	13
*S. divinum*	*Sphagnum*	9	9	0	18
*S. fimbriatum*	*Acutifolia*	4	4	3	11
*S. flexuosum*	*Cuspidata*	9	3	2	14
*S. fuscum*	*Acutifolia*	8	9	2	19
*S. lindbergii*	*Cuspidata*	9	3	2	14
*S. platyphyllum*	*Subsecunda*	8	4	0	12
*S. riparium*	*Cuspidata*	8	5	1	14
*S. squarrosum*	*Acutifolia*	9	8	2	19
*S. subnitens*	*Acutifolia*	8	9	2	19
*S. tenellum*	*Cuspidata*	12	6	2	20

Note.—Number of samples collected from each of the allopatric populations for each of the 12 studied species.

### Genetic Differentiation among the Species

We first explored the relationship of the species using principal component analysis (PCA) performed on the genetic covariance matrix among all individuals based on 16.3 million (M) biallelic sites. The PCA demonstrated a considerable level of interspecific differentiation with most of the species forming distinct point clusters in the space of the first three principal components (Proportion of total variance explained: *PC1* 12%, *PC2* 10%, *PC3* 10%, fig. 1). Individuals of the three species, *S. capillifolium*, *S. fuscum*, and *S. subnitens*, that appeared unresolved in the space of *PC1*–*PC3*, were well-separated in a PCA carried out only on the subset of these individuals (supplementary fig. S2, Supplementary Material online).

We further estimated individual ancestry assignment and admixture using ADMIXTURE (Alexander et al. 2009) to detect recent introgression among the species. The analysis based on 23,560 independent SNPs both supported *K *=* *12 (cross-validation error 0.06, supplementary fig. S3, Supplementary Material online) and did not reveal either evidence for strong genetic structure within or substantial admixture between species, with the exception of one *S. flexuosum* individual whose genome may be shared with *S. tenellum* (30%) (fig. 1*D*).

**Fig. 2.**
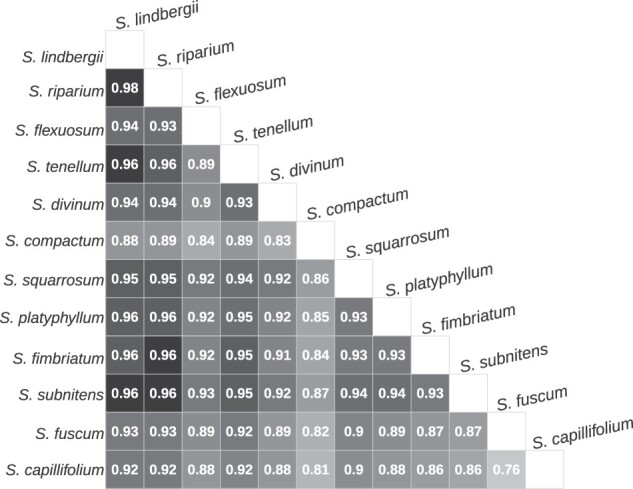
Global pairwise weighted *F*_ST_ among the *Sphagnum* species investigated.

To estimate the level of differentiation among the species, we calculated genome-wide *F*_ST_ based on 121 ± 10 M (SD) biallelic sites for each species pair taking into account genotyping uncertainty (see Materials and Methods). The values of *F*_ST_ vary from 0.75 to 0.98 among the species pairs (fig. 2). Together with the results of the PCA and ADMIXTURE analysis, this suggests that all studied species are highly differentiated from one another which is in accordance with other studies showing high level of genetic distinctiveness in *Sphagnum* (Shaw, Shaw, Johnson, et al. 2015; Kyrkjeeide et al. 2016; Yousefi et al. 2017, 2019).

### Phylogenetic Relationships between the Species

**Fig. 3.**
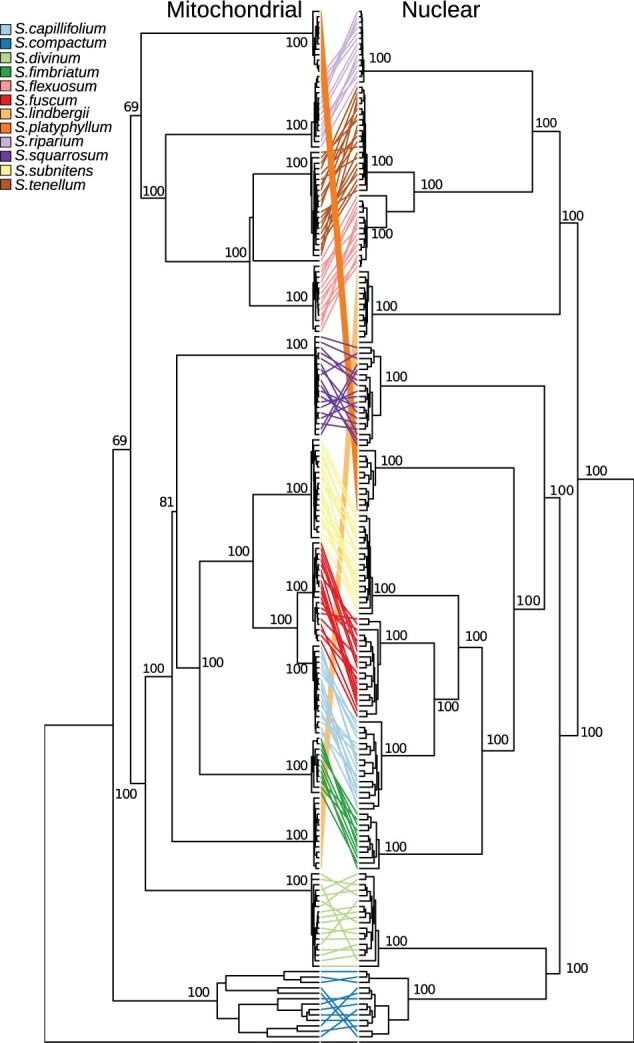
Phylogenetic relationships among the species inferred using concatenated genetic markers (mitochondrial and nuclear dendrograms). The colored lines connect samples of the same species on the two dendrograms, the color code is shown on the left.

To infer the phylogenetic relationship among the species, we first used the filtered nuclear SNP data set (455K SNPs, see Materials and Methods) to perform a maximum-likelihood analysis in RAxML. We also carried out the very same analysis on the chloroplast (1.9 K SNPs) and on the mitochondrial (1 K SNPs) SNP data sets. Organellar genomes are believed to be maternally inherited and nonrecombining, as shown in some peatmosses (Natcheva and Cronberg 2007). Assuming no genetic exchange among taxa and complete lineage sorting, nuclear, and organellar phylogenies should resolve the very same relationships. However, the analyses of organellar and nuclear SNPs provided very well-resolved, but topologically conflicting, phylogenies with each species forming a strongly supported monophyletic clade (fig. 3). The relationships inferred using plastid and mitochondrial markers are congruent to the most comprehensive organellar-based phylogenies of the genus (Shaw et al. 2016, 2019) and to each other, except that the admixed *S. flexuosum* individual (ND4) is resolved either within *S. flexuosum* or *S. tenellum* in the chloroplast-based and in the mitochondrion-based phylogeny, respectively (in red on supplementary fig. S5, Supplementary Material online). The nuclear and organellar markers led to conflicting phylogenies regarding both the relationships among the subgenera and the placement of individual species, namely, *S. lindbergii*, *S. compactum*, *S. divinum*, *S. platyphyllum*, and *S. squarrosum* (fig. 3).

In all phylogenies, there was no evidence for geographical structure within species, with the exception of *S. compactum*, for which samples from Norway, Germany, and Austria form distinct clades (supplementary fig. S5*D*, Supplementary Material online). This suggests weak genetic structure within peatmoss species across the sampled distribution, corroborating previous observations in many *Sphagnum* species (Szövényi et al. 2008, 2012; Stenøien et al. 2011; Szurdoki et al. 2014; Mikulášková et al. 2015; Shaw, Shaw, Stenøien, et al. 2015; Kyrkjeeide et al. 2016).

### Coalescent-Based Analysis

**Fig. 4.**
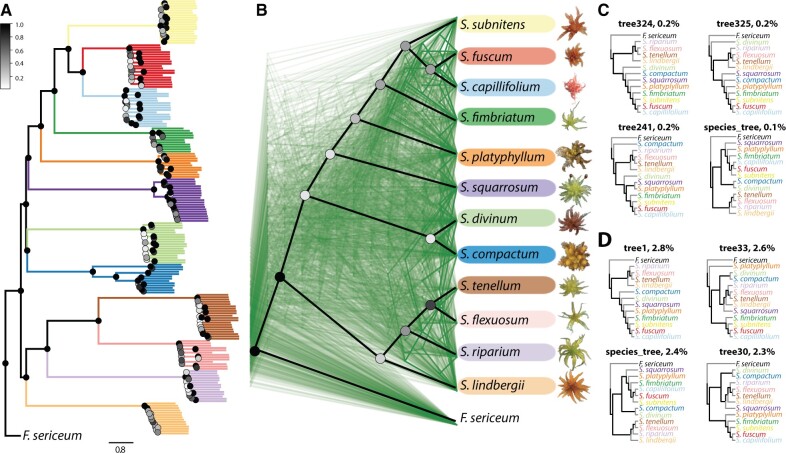
The coalescent- and sliding-window based phylogenies. (*A*) The coalescent-based species tree. The node color represents ASTRAL local posterior support according to the scale shown on the left. Color of branches refers to the species as on (*B*), length of the branches is in coalescent units as shown at the bottom. (*B*) Cladograms of the coalescent-based species tree (heavy black lines) and of 500 gene trees (in green) randomly sampled from 988 inferred gene trees. Each species is downsampled to one sample (supplementary table S1, Supplementary Material online). The node color represents node recovery (concordance factor), that is, the fraction of all gene trees recovering a particular node, according to the scale shown on the left. Photos of capitula were taken by Kjell Ivar Flatberg. (*C*) The most common topologies in gene trees and (*D*) in 100-kb sliding-window trees, the number represents the percentage of trees with the given topology.

It is well known that phylogenetic analysis using concatenated data sets does not take into account the stochasticity of the coalescent process and often fails to recover the true species tree (Kubatko and Degnan 2007). Therefore, we also used a coalescent-based phylogenetic method to infer the species tree from a set of gene trees by explicitly taking into account the inherent stochasticity associated with the coalescent process (Rabiee et al. 2019). Our coalescent-based analysis of 988 genes (1.7 ± 1.2 kb [SD], supplementary table S4, Supplementary Material online) recovered the very same highly supported species tree as our RAxML analysis with an ASTRAL quartet score of 79% (fig. 4*A*). This suggests that the incongruence between the organellar and nuclear phylogenies is real and not simply due to phylogenetic error using concatenated data sets (Wang and Hahn 2018).

To explore the distribution of discordance across the species tree, we calculated the concordance factor, which corresponds to the number of gene trees recovering a particular node. The results show that the deeper nodes describing the split among taxa and taxon groups are supported by only a small fraction, whereas the monophyly of all species is recovered by the majority of the gene trees (fig. 4*B* and supplementary fig. S6*B*, Supplementary Material online). We also found that the poorly-supported nodes corresponded to those causing incongruence between the nuclear- and the organellar-based phylogenies. In particular, only 12% of the trees recover the node uniting all species in the clade sister to the *Cuspidata* species (table 1), and only 12% of the trees resolve *S. divinum* and *S. compactum* as sister species. Most of the trees recover the species tree branching order within *Cuspidata* and *Acutifolia* species, whereas the positions of *S. squarrosum* and *S. platyphyllum* within the *Acutifolia* clade were recovered in 13% and 28% of the trees, respectively. Similarly, the placement of *S. lindbergii* was supported in only 20% of the trees. We observed that the branch length at the node was positively correlated to the node recovery (*r*_S_* *=* *0.77, *P* < 0.0001, supplementary fig. S6*A*, Supplementary Material online), and, despite low recovery by the gene trees, all deep nodes received very high posterior support in ASTRAL (fig. 4*A*). We also compared the among-species topology of each gene tree to the species tree topology and found that 99% of the gene trees had different topologies, and only one of 988 gene trees had the species tree topology (fig. 4*C*). Short branches at the deeper nodes suggest ILS as the cause of the observed incongruences, but gene flow among the species can also distort the phylogenetic signal across the genome.

Because our coalescent-based analysis was based on 988 genes randomly sampled across the genome, we extended our analysis to many more sites by estimating phylogenetic trees in 100-kb nonoverlapping windows across all genomic scaffolds longer than 2 Mb. In total, the resulting trees contained 141 M distinct site patterns and 19 M parsimony informative sites compared with 1.5 M and 118 K, respectively, found in the gene trees (supplementary tables S5 and S4, Supplementary Material online). Similarly, to the pattern we observed with the gene trees, only 2.4% out of the 650 different topologies identified in the 1,774 sliding window trees matched the species tree topology (fig. 4*D*). Concordance factor estimates confirmed our previous estimates based on genes, nevertheless, the node recovery was higher for the sliding window trees than for the gene trees (31.5% [SE = 2.4] vs. 12.7% [SE = 1.7], respectively, Wilcoxon rank sum test, *P* < 0.0001).

### *D*-Statistics

**Fig. 5.**
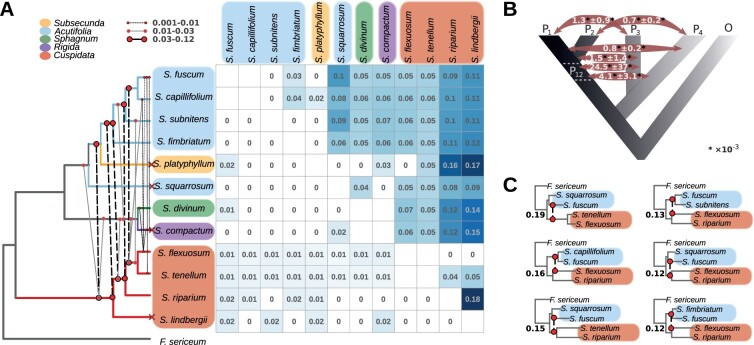
Tests for introgression. (*A*) Mean pairwise *D* per species pair (upper diagonal) and the mean total proportion of introgressed loci per species pair inferred through the QuIBL analysis (lower diagonal). Empty squares correspond to the pairs that have not been tested since they are sister species in our data set, and 0 values correspond to nonsignificant values. The nuclear-based cladogram is shown on the left, red cross symbols designate the species that are placed in disagreement with the plastid-based phylogenies. The color of boxes and branches represents the subgenus as shown on the top left. The cladogram includes lines schematically representing interspecific introgression events based on summarized results of the *D*_FOIL_ analysis. The color and shape of the lines indicate the average portion of windows supporting introgression between the branches as shown on the top. (*B*) Schematic summary of results of *D*_FOIL_ analysis on a five-taxon phylogeny with four in-group taxa (P_1_−P_4_) and an outgroup (O), P_12_ is an ancestral branch. The numbers correspond to the proportion of introgressed windows for the corresponding type of introgression (P_1_⟺P_3_, P_2_⟺P_3_, P_2_⟺P_4_, P_12_⟺P_3_, P_12_⟺P_4_) averaged in all tested five-taxon topologies followed by its standard deviation. (*C*) Fixe-taxon phylogenies with the highest proportion of introgressed windows inferred with the *D*_FOIL_ analysis. The phylogenies include lines, which represent introgression events as in (*A*), the numbers represent the corresponding proportion of windows showing ancient introgression to the total number of windows analyzed for the phylogeny. Color of species names highlights represents the subgenus the species belongs to as in (*A*).

To assess whether the observed phylogenetic incongruences are mainly due to interspecific gene flow, we calculated Patterson’s *D*-statistic (hereafter referred to as *D*) implemented in ANGSD, which uses the ABBA-BABA test for introgression among a quartet of species (Soraggi et al. 2018). To carry out the test, we used all consistent with the nuclear phylogeny quartet topologies as (((P_1_, P_2_),P_3_),outgroup) to test for evidence of excess of derived sites shared by P_3_ and P_1_ or P_2_ versus shared by P_1_ and P_2_ defining *F. sericeum* as an outgroup (supplementary table S6, Supplementary Material online). Three species pairs could not be tested (*S. tenellum* and *S. flexuosum*, *S. capillifolium* and *S. fuscum*, *S. compactum* and *S. divinum*, designated with empty squares on fig. 5) since they are sister species in our data set. Our block jack-knife analysis supports the view that *D* is significant in many of the triplets (80%, 175 out of 220 triplets, *P* < 0.002, supplementary table S6, Supplementary Material online). Our estimates of *D* and number of sites assessed varied widely depending on the third species in the triplet (P_1_ or P_2_, supplementary fig. S7, Supplementary Material online), thus a mean value of absolute *D* for a species pair was calculated from all triplets in which these species had significant values of *D*. The absolute *D* was significant in most of the pairwise species comparisons (81%, 51 out of 63 pairwise comparisons based on 22–41 K sites, supplementary table S7, Supplementary Material online), and varied from 0.02 to 0.18 (fig. 5). For some species pairs, *D* was not significantly different from zero (fig. 5). Our analysis was insensitive to the size of the genomic window used for the analysis (1 and 5 Mb, supplementary fig. S7*E*, Supplementary Material online). The species showing incongruent placement across the phylogenies have low, yet significant, values of *D* with species of subgenus *Cuspidata* and *Acutifolia* (fig. 5), which suggest that gene flow might have contributed to the observed phylogenetic discordance.

Assuming that gene flow is on-going or recent, it might be restricted to closely-related species as reproductive barriers accumulate with time since divergence (Coyne and Orr 2004). Therefore, one might expect *D* to be negatively correlated with differentiation between the species. In contrast, *D* was positively correlated with *F*_ST_ (*r*_S_* *=* *0.32, *P* = 0.02, supplementary fig. S7*D*, Supplementary Material online). Given that our ADMIXTURE analysis did not reveal recent genomic exchange among the species, this suggests that the introgression inferred with *D* happened earlier in the diversification process, and likely among the ancestral lineages (see this hypothesis explicitly addressed under “Testing for Ancient Introgression” below).

We additionally performed tests for admixture using 23,560 independent SNPs with TreeMix (Pickrell and Pritchard 2012) and calculated the *f*_3_ statistics (Keinan et al. 2007). We did not find evidence for recent gene flow with the *f*_3_ statistics (supplementary table S8, Supplementary Material online), whereas the results of the TreeMix analysis were ambiguous with different models being equally supported. Namely, the inferred migration events were inconsistent, and the likelihood of the estimated tree model did not greatly differ among replicates with the same number of migration events allowed and among the best supported replicates (supplementary fig. S8, Supplementary Material online). This might suggest a complex rather than tree-like evolutionary history of the studied species (Foote and Morin 2016).

### QuIBL Analysis

Because we observed that phylogenetic discordance can be potentially associated with both ILS and introgression, we made use of a recently developed tree-based method, QuIBL (Edelman et al. 2019), to differentiate between these two processes. QuIBL estimates the distribution of internal branch length in discordant topologies for triplets of species, and then calculates the likelihood that this distribution corresponds to the model with introgression and ILS or with ILS only. QuIBL therefore only estimates postspeciation gene flow for a set of three species at a time. For the analysis, we kept one sample with the highest coverage per species and, since the method is sensitive to recombination (Edelman et al. 2019), generated 3,195 trees (supplementary table S11, Supplementary Material online) based on small windows (2 kb) considerably distant from each other (20-kb steps). Because *S. compactum* showed pronounced geographic structure, we used samples from two different populations in the analysis and obtained the same results for both samples (not shown). Three closely-related species pairs could not be tested as they are sister species in our data set (marked with empty squares on fig. 5*A*).

The QuIBL analysis revealed that only 22% of the tested triplets showed significant evidence for introgression (48 of 220 triplets, ΔBIC > 10, supplementary table S9, Supplementary Material online). For the rest of the triplets, adding introgression did not improve the fit of the ILS-only model. Furthermore, we found that only 0.29% of loci supported discordant topologies and were introgressed (supplementary fig. S9, Supplementary Material online), suggesting limited overall interspecific introgression among the studied species (fig. 5*A* and supplementary table S10, Supplementary Material online). QuIBL analysis suggests ILS, rather than postspeciation introgression, is the main factor behind phylogenetic discordance among the species.

### Testing for Ancient Introgression

Our *D*-statistic-based analysis revealed significant, albeit relatively low, levels of recent gene flow among species. Nevertheless, the degree of introgression between taxa (*D*-statistic) was positively correlated with between-species genetic differentiation (*F*_ST_) suggesting that ancient introgression among the ancestors of extant subgenera could have been more frequent than postspeciation gene exchange. Therefore, we further explored this hypothesis with the *D*_FOIL_ statistic (Pease and Hahn 2015). The method uses a group of four species and an outgroup as (((P_1_, P_2_),(P_3_, P_4_)),O) to quantify introgression among nonsister tips, as well as ancient introgression. *D*_FOIL_ performs well in the presence of ILS, and at low rates of introgression (Pease and Hahn 2015). We used one sample per species with the highest coverage (supplementary table S1, Supplementary Material online), *F. sericeum* as the outgroup, and computed *D*_FOIL_ for all five-taxon combinations consistent with the nuclear phylogeny (supplementary table S12, Supplementary Material online) in 100-kb nonoverlapping windows across the scaffolds longer than 1 Mb (Pease and Hahn 2015). *D*_FOIL_ analysis showed that 98% of five-taxon phylogenies (180 out of 183, supplementary table S12, Supplementary Material online) showed evidence for introgression. In accordance with the results of the traditional *D*-statistic and QuIBL, the analysis revealed very low levels of postspeciation gene flow among the extant species (<0.1% of all windows, supplementary table S13, Supplementary Material online, and fig. 5*B*), whereas up to 19% of the windows showed evidence of ancient introgression between (P_1_, P_2_) and P_3_, and up to 1.3% between (P_1_, P_2_) and P_4_ (supplementary table S13, Supplementary Material online and fig. 5*B*). We found 11 five-taxon phylogenies which had >10% windows introgressed from (P_1_, P_2_) into P_3_ (fig. 5*C*). In agreement with the *D*-statistic test, these results support genomic exchange between the ancestor of the species of subgenus *Cuspidata* (*S. flexuosum*, *S. riparium*, *S. tenellum*, *S. lindbergii*) with the ancestor of the species of subgenus *Acutifolia* (*S. fimbriatum*, *S. subnitens*, *S. fuscum*, *S. capillifolium*), as well as with the ancestors of *S. compactum* and *S. divinum*.

The length of consecutive introgressed regions can be used to infer the relative timing of introgression (Barlow et al. 2018; Moodley et al. 2020; Westbury et al. 2020). Assuming recent introgression, longer stretches of introgressed genomic segments are expected to be found in the genome, which with time get broken into smaller pieces due to recombination (Węcek et al. 2017). We therefore investigated if the windows showing any evidence for introgression formed consecutive regions. We found that most of the introgressed windows were singletons, with occasional blocks of two to four consecutive introgressed windows (supplementary fig. S10 and table S14, Supplementary Material online). For instance, in the five-taxon phylogeny with the highest number of introgressed windows (fig. 5*C*), 87% of the windows were singletons, and 10% were arranged in blocks of two consecutive windows. Taken together, these results strongly support the hypothesis of ancient introgression among the ancestral *Sphagnum* species.

## Discussion

In this study, we focused on the genome-wide pattern of phylogenetic discordance and its relationship to interspecific introgression and ILS in 12 peatmoss species, representing all subgenera within the genus, sampled at different geographical scales. Our analyses show that all species are highly genetically differentiated from one another, and most show no evidence for population genetic structure across the sampled distribution. At the same time, phylogenetic discordance was common both across the genome and between the organellar- and nuclear-based phylogenies. We found evidence for ancient introgression among the ancestral populations likely followed by ILS, whereas levels of postspeciation gene flow were surprisingly low. Our analyses show that ILS, and not extensive introgression, has mainly been responsible for shaping the genomic landscape of diversification in this diverse group of plants. In the following paragraphs, we discuss how these two processes may have shaped the diversification of *Sphagnum* mosses.

### Genomic Signatures of Rapid Radiation

*Sphagnum* is known for a recent rapid burst of diversification 7–20 Ma associated with the Miocene cooling, which presumably triggered adaptation to diverse, novel habitats (Shaw, Devos, et al. 2010). Rampant radiation should lead to short coalescent times and ILS among newly formed lineages (Whitfield and Lockhart 2007). Consequently, phylogenetic studies of extant lineages in such a case would infer hemiplasy (Avise and Robinson 2008), that is, topological incongruences between the species tree and gene trees, irrespectively of type, quality, and quantity of the markers used in the analysis (Pease et al. 2016; Wang and Hahn 2018). This is precisely the pattern we observed in both gene and sliding-window tree analyses, as well as in organellar genome phylogenies in peatmosses. Additionally, rapid speciation often results in the anomaly zone phenomenon, in which the true species tree topology is difficult to resolve for inferences involving more than four taxa (Degnan and Rosenberg 2009; Suh et al. 2015). This explains the numerous conflicting topologies of the gene and sliding window trees and the low recovery of deep nodes, but strong support of the species tree topology inferred with ASTRAL, which is robust to the presence of the anomaly zone (Allman et al. 2011) as it takes a quartet of leaves at a time under the multispecies coalescent model (Mirarab et al. 2014). Similar extent and distribution of discordance among loci in the genome and across the nodes in the species tree has been found in rapidly radiated groups, both recently diverged, such as in wild tomatoes (Pease et al. 2016) and cichlid fishes (Irisarri et al. 2018), and at a deeper timescale, such as in neoavian birds (Suh et al. 2015). Altogether, rapid diversification of peatmosses has likely significantly contributed to the extensive phylogenetic incongruence observed, which is in line with findings of previous studies investigating the genomic signatures of rapid radiations in various groups of organisms.

When time after divergence increases, so does the probability that parallel evolution has occurred in the diverged lineages (Suh et al. 2015). Therefore, one could argue that some or most incongruences are due to parallel evolution rather than ILS. Considering that in *Sphagnum* the last diversification peak is relatively recent, it is less likely that the observed deep-node incongruences occur due to homoplasy rather than hemiplasy derived from ILS (sensu Suh et al. 2015). Although the radiation of peatmosses was clearly sudden (Shaw, Devos, et al. 2010; Devos et al. 2016), there is some uncertainty about its timing, which might have been much older than previously thought (Shaw et al. 2019). Nevertheless, no alternative date has been suggested, and the strong negative relationship between incongruence at a node and the internode length in our gene tree concordance factor analysis further supports the idea that high levels of ILS, and not homoplasy, substantially contribute to the observed genome-wide phylogenetic signal (Zwickl et al. 2014; Pease et al. 2016; Irisarri et al. 2018).

### Recent Introgression or Incomplete Lineage Sorting?

Based on the results of the phylogenetic analyses, we expect ILS to be the main factor explaining the observed phylogenetic discordance. However, these analyses do not differentiate between ILS and introgression, both of which can create similar phylogenetic signals (Fontaine et al. 2015). Considering that all species are highly supported as monophyletic clades and that no signs of recent admixture were found in the ADMIXTURE analysis, a potential explanation is that although interspecific introgression is currently constrained by strong reproductive isolation among the species, gene flow was more prominent in the past, during the speciation process, facilitating *Sphagnum*’s known rapid diversification into diverse habitats (Shaw, Devos, et al. 2010; Devos et al. 2016). It has been suggested that ancient admixture of ancestral variation is a powerful means for rapid radiations to occur (Marques et al. 2019). The results of our introgression and QuIBL tests support this idea.

We did not find significant evidence for introgression using ADMIXTURE and the *f*_3_ statistics, which are suited to detect recent introgression (Alter et al. 2017). Levels of postspeciation interspecific gene exchange inferred with QuIBL and *D*_FOIL_ were very low, whereas the *D*-statistic showed considerable introgression among species pairs with deeper nodes and generally low values in more recent species pairs. For instance, the *D*-statistic shows that *S. lindbergii* and *S. riparium* share 4–18% of the derived sites with other studied species. *Flatbergium sericeum*, which was used as an outgroup in our *D* test, has a smaller genome size and number of chromosomes than *Sphagnum* (Shaw, Cox, et al. 2010) and represents a family of non-*Sphagnum* peatmosses that diverged from *Sphagnum* peatmosses approximately 34–105 Ma (Shaw, Devos, et al. 2010). Using a distantly related outgroup does not in itself affect the robustness of *D* (Zheng and Janke 2018), but our estimates in this test are tied to the sites where the outgroup sequence reads can be mapped to the *S. angustifolium* reference genome, likely covering the more conserved part of the genome. Consequently, the observed *D* values likely represent retained traces of ancient introgression events among ancestors of the extant species followed by genetic drift or divergent selection. This is further supported by the results inferred with *D*_FOIL_ for different combinations of the species, which suggest that gene flow likely happened between the ancestors of the species from subgenus *Cuspidata* and the ancestors of other subgenera. Despite that *D*_FOIL_ cannot infer introgression between the two ancestral lineages in a five-taxon topology (Pease and Hahn 2015), the results inferred using different combinations of species from these two subgenera can only be explained by a deep introgression event, which should have happened early in the diversification process, followed by differential retention of ancestral polymorphism in the studied species due to subsequent genetic drift and/or divergent selection. A similar pattern has been recently described in rhinoceros, where gene flow between the ancestral lineages ceased within 2 My after initial divergence, but resulted in false-positive signatures of introgression among the subsequently diverged subspecies due to random coalescent processes (Moodley et al. 2020).

Alternatively, the *D* and *D*_FOIL_ tests may have inferred false-positive values due to certain population structure in the ancestral lineage or differences in the effective population size among the lineages (Zheng and Janke 2018), which is plausible given the recent bottleneck documented in extant peatmoss species (Thingsgaard 2001; Kyrkjeeide et al. 2012; Yousefi et al. 2017). *D*_FOIL_ can incorrectly infer ancient introgression between (P_1_, P_2_) and P_3_ or P_4_ if introgression happened shortly after the split of (P_1_, P_2_) (Pease and Hahn 2015) or if P_3_/P_4_ exchanged genes with both P_1_ and P_2_ at equal rate since their split (Fontaine et al. 2015). In our case, however, the majority of different five-taxon phylogenies demonstrated significant and consistent signals for ancient introgression deep in the species phylogeny. In addition, the prevalence of singleton introgressed windows we observe with *D*_FOIL_ also suggests that these windows indeed represent signatures of ancient, rather than recent, introgression (Barlow et al. 2018; Moodley et al. 2020; Westbury et al. 2020).

We have to note that recent introgression might have been underestimated in our tests for various reasons detailed below. Introgression from a “ghost” unsampled population or difference in population sizes among the species can also bias *D* (Zheng and Janke 2018) and *D*_FOIL_ estimates (Pease and Hahn 2015) which are possible sources of errors we could not account for. The QuIBL analysis can also provide biased estimates: it will likely overestimate introgression if the windows used for the analysis contain recombination breakpoints (Edelman et al. 2019). We took this into account in our test by choosing a narrow window size of 2 kb to minimize the risk of including many recombination breakpoints. Accordingly, we believe that our QuIBL estimates are reliable. Unlike the *D*-statistic, QuIBL provides robust estimates when all three species in the triplet hybridize, which likely occurs, but will not lead to further biases (Edelman et al. 2019). On the other hand, differential loss of gene copies after the whole-genome duplication events known in *Sphagnum* (Devos et al. 2016) might lead to longer internal branches. If this is the case, QuIBL would overestimate gene flow (Edelman et al. 2019). In line with this, we found higher levels of postspeciation gene flow with QuIBL than with *D_FOIL_*. The proportion of differentially retained loci is unknown, hence their effect on gene flow estimation should be addressed in future studies of *Sphagnum* genomics using de novo assembly.

Although our results imply that postspeciation gene flow is minimal, they do not exclude the possibility of significant interspecific gene-flow among selected peatmoss species. *Sphagnum* includes about 300 species distributed worldwide (Michaelis 2019), and we did not sample very closely related species (sister species) in this study, and could not test the most closely related species pairs in most of our introgression analyses. It is therefore possible that recent, and more significant interspecific introgression may take place between very closely related species pairs that were not included in this study. Hence, our finding of minimal contemporary postspeciation gene flow among peatmoss species applies only to the set of species used in this study. In turn, the single admixed individual identified in our study, resulting from admixture between two sister species (*S. flexuosum* and *S. tenellum*), could be an F_1_ hybrid, which does not necessarily imply ongoing introgression between these species, but simply hybridization. Considering that the known widespread interspecific hybridization in *Sphagnum* is mostly happening in the form of allopolyploidization (reviewed in Meleshko et al. 2018), our findings may indicate that the group has evolved strong reproductive barriers, which prevent homoploid hybridization from translating into substantial introgression.

Ancient introgression and subsequent ILS, together with very limited postspeciation introgression, agree very well with all our findings and with the extensive discordance we identified in our phylogenomic analyses. This scenario explains the inconsistent placement of *S. lindbergii*, *S. compactum*, *S. divinum*, *S. platyphyllum*, and *S. squarrosum* within the gene and sliding-window phylogenies, as well as the incongruences among the nuclear and the organellar phylogenies. It is, however, beyond the scope of this study to determine the relative impact of these two processes on the early diversification process in this group. There were 53 triplets in our QuIBL analysis for which the most common topology (supported by the highest number of trees) did not correspond to the species tree topology (supplementary table S11, Supplementary Material online). In particular, for the triplet (*S. lindbergii*, (*S. divinum*, (*S. squarrosum*))), all three possible topologies were nearly equally supported by the trees. Other such triplets consistently showed discordance in placement of *S. squarrosum*, *S. compactum*, and *S. divinum* relative to the backbone *Acutifolia* species (*S. capillifolium*, *S. fuscum*, *S. subnitens*, *S. fimbriatum*) and to *Cuspidata* species. None of these triplets showed significant evidence for postspeciation introgression, and average levels of ancient introgression inferred with *D*_FOIL_ were moderately low (fig. 5*B*). These are the same incongruences we detected in our gene and sliding-window trees analyses, suggesting that the species tree topology might itself have originated from ILS (Edelman et al. 2019). With this high level of ILS, a bifurcating tree might therefore be an oversimplification of the true evolutionary history of this rapidly radiated group.

## Conclusions

Our analyses suggest the following hypothesis about the evolutionary history of peatmoss. When *Sphagnum* started to diversify, effective population sizes were large and gene flow extensive among the emerging species, which resulted in plenty of shared polymorphism among species. This great diversity was then sorted out following rapid diversification, triggered by whole-genome duplication (Devos et al. 2016), into diverse habitats, newly formed as a consequence of rapid climate change (Shaw, Devos, et al. 2010). Finally, reproductive isolation and/or restricted gene flow gave rise to the current species diversity.

Our findings demonstrate that rapid radiation creates a phylogenomic pattern in bryophytes similar to that observed in angiosperms, which corroborates the suggested idea of universality of evolutionary processes among land plants (Medina et al. 2018). In contrast to many recently rapidly radiated, actively hybridizing groups, postspeciation gene flow is not prominent in creating phylogenetic discordance in *Sphagnum*, at least not in the species studied here. Based on our results, the evolutionary history of peatmoss might be too complex to be modeled as a simple bifurcating tree, and reconstructed using a single type of genetic markers. This needs to be taken into account in further studies of this and other rapidly radiated bryophyte groups.

## Materials and Methods

### Sampling

We sampled 12 common haploid species with no known hybrid origin and contrasting life-history traits that represent different subgenera within *Sphagnum* (table 1). The sampling was carried out in three metapopulations from central Norway, Austria, and Germany (fig. 1). For each of the 12 species, we sampled two to three populations in each of two European regions and, for most species, one population in Germany. Two to four individuals were collected at each population for a total of 11–20 individuals per species (a total of 190 individual sampled shoots, table 1). Vegetative reproduction is common in peatmosses, so to avoid sampling clones within possible mating distance, we collected only conspecific shoots growing approximately 1 m apart. The accessions and the voucher specimens were air-dried and deposited at the Trondheim Herbarium (TRH). Additionally, we included a sample of *Flatbergium sericeum* (Müll. Hal.) A.J. Shaw from the TRH to use as an outgroup in various analyses. For a list of voucher specimens, see supplementary table S1, Supplementary Material online.

### DNA Extraction

DNA was extracted from cleaned dried capitula tissue and fragmented to a mean length of 400 bp as described in detail in supplementary SMM1, Supplementary Material online.

### Library Preparation and Sequencing

Our study provides the first investigation using whole-genome sequencing in peatmosses. Therefore, we tested the performance of the library preparation and sequencing method on a subset of 11 samples (one accession per species). Individual whole-genome DNA libraries were prepared as described in detail in supplementary SMM2, Supplementary Material online and sequenced at the Functional Genomics Center Zurich (FGCZ, Switzerland) on a single lane of an Illumina HiSeq 4000 in 150-bp paired-end mode. The rest of the libraries were prepared and pooled into 16 pools based on the estimated per-library endogenous content as described in detail in supplementary SMM3, Supplementary Material online. The negative library build and indexing PCR controls were included into one of the pools. Sequencing was performed at the Genomics Core Facility, Faculty of Medicine, NTNU (Trondheim, Norway) on two flowcells of an Illumina HiSeq 4000 in 150-bp paired-end mode.

### Sequencing Data Processing

The raw sequencing reads were processed using the Paleomix pipeline v1.2.13.4 (Schubert et al. 2014). Adapter contamination was trimmed using AdapterRemoval v2.2.0 (Schubert et al. 2016), and trimmed reads shorter than 25 bases were discarded. The remaining reads were mapped to a reference genome assembly of *Sphagnum angustifolium* (formerly *fallax*, v0.5, DOE-JGI, http://phytozome.jgi.doe.gov/, last accessed March 1, 2021), using the mem algorithm of BWA v0.7.15 (Li and Durbin 2009). Aligned reads with a mapping quality (MAPQ) score below 30 were discarded. PCR duplicates were marked with PicardTools v2.9.1 (http://broadinstitute.github.io/picard, last accessed March 1, 2021). We performed realignment around indels using the Genome Analysis Toolkit (GATK) v3.7 (McKenna et al. 2010) to reduce the number of alignment artifacts, and validated the resulting bam files with PicardTools v2.9.1. We used SAMtools v0.1.19 for sorting, converting, and generating summary statistics for the bam files (Li et al. 2009). The raw reads were also aligned to *Sphagnum fallax* chloroplast and mitochondrion genome sequences (GenBank accession codes KU725463 and KU725501, respectively) in the same manner as described above.

### SNP Calling and Filtering

The variants were called with “-ploidy 1” tag using the GATK v3.7 “HaplotypeCaller” for each sample separately. Next, the samples were divided into the sets of 20–25, and each set was genotyped with the GATK v3.7 tool “GenotypeGVCF.” Following the best practices pipeline (Van der Auwera et al. 2013), we extracted SNPs from the call sets and performed hard-filtering with the recommended parameters: QualByDepth <2.0, FisherStrand >60.0, RMSMappingQuality <40.0, MappingQualityRankSumTest < −12.5, ReadPosRankSumTest < −8.0. The SNPs meeting any of these criteria were excluded from the data set. We tested different filtering parameters for missingness and depth using VCFtools (Danecek et al. 2011). As the data set included 12 different species, strict missingness criteria led to a dramatic decrease in the number of SNPs. Therefore, we kept the SNPs that were present in at least 20% of all individuals, also filtering these for minimum mean depth of 5, maximum mean depth of 100, and minimum minor allele frequency of 0.05. The resulting data set is referred to as the filtered nuclear SNP data set. Plastid and mitochondrial genome alignments were treated in the same manner, except that we kept the SNPs that were present in 100% or in at least 50% of all individuals for the chloroplast and mitochondrial alignments, respectively, and no maximum mean depth was used to filter the SNPs. The resulting data sets are referred to as the chloroplast SNP data set and the mitochondrial SNP data set, respectively. When applying the software sensitive to linkage between the SNPs, we randomly selected one SNP per 2,000 bp from the filtered nuclear SNP data set. This data set is referred to as the thinned SNP data set. A summary of the number of genetic markers used in each of the analyses described below can be found in supplementary table S3, Supplementary Material online. Given that the ploidy level was set to 1, no heterozygous variants were called. Therefore, we calculated the percentage of potentially heterozygous variants, which could have potentially been called from mismapped paralogous genomic regions, as described in detail in supplementary SMM6, Supplementary Material online. We found a very low number of sites showing heterozygous signals in our SNP data set (mean 0.43% ±0.18% SD across samples; supplementary SMM6 and fig. S11, Supplementary Material online), which is in line with our assumption that mismapping of paralogous copies do not have a considerable effect on the number of SNPs called.

### Principal Component Analysis

We computed the covariance matrix between individuals using ANGSD v0.931-8-g1ed4245 by sampling a random base from each individual at each position for biallelic sites (Korneliussen et al. 2014) to exclude bias introduced by differences in sequencing depth. First, we performed per-base alignment quality (BAQ) computation implemented in ANGSD to adjust quality scores around indels in the mapped reads used as an input (Li 2011), and adjusted MAPQ score to 50 for reads with excessive mismatches. Then, those reads with poor quality (flag≥256), low MAPQ score (≤30), low base quality score (≤20), or with unmapped mate and secondary alignments were discarded. These reads filtering procedures were also used for other analyses performed in ANGSD and are hereafter referred to as “read quality filtering in ANGSD.” Minor alleles were inferred by picking the two most frequently observed bases across individuals (Li et al. 2010). Then, sites were filtered based on minimum minor allele frequency (≥0.05) and sample size (≥⅓ of individuals). Following (Patterson et al. 2006), we generated eigenvectors for the covariance matrix in R and performed a Tracy-Widom test to determine the significance of the eigenvalues using the package “AssocTest” (Wang et al. 2020).

### Admixture Analysis

ADMIXTURE v1.3.0 was used to estimate individual assignment and admixture (Alexander et al. 2009). We excluded the outgroup from the thinned SNP data set and used bcftools (http://samtools.github.io/bcftools/, last accessed March 1, 2021) and PLINK v1.90b6.9 (Chang et al. 2015) to convert the VCF into a binary PLINK file to be used in ADMIXTURE. Due to limitations of PLINK, we kept the 95 longest scaffolds during the conversion that equal to 63% (249.6 M bases) of the total length of the reference. To infer the best number of *K*, 10-fold cross-validation procedure was used, testing *K *=* *1 to *K *=* *16. For each *K*, 20 independent runs were performed, and the mean cross-validation error among all replicates for each *K* was calculated and compared with identify the replicate with the lowest error.

### Test for Ancestral Admixture

In order to reconstruct major migration events in the group, we performed a TreeMix v1.13 (Pickrell and Pritchard 2012) analysis which uses allele frequency data to reconstruct the relationships among the species as a bifurcating ML tree that corresponds to the estimated degree of genetic drift among the species. We calculated allele frequencies per species with PLINK using the same input file as for the ADMIXTURE analysis above, and imported these into TreeMix. TreeMix was run with bootstrap without incorporating migration and with allowing from one to five migration events. For each scenario, 100 independent runs were performed, and runs with the highest log likelihood for each scenario were selected. From the covariance matrix estimated from the data in the best runs, we calculated the total standard error and the amount of variance in species relatedness explained by the model using an R script by Card (2015). We also used TreeMix to calculate the *f*_3_ statistics on the same data set (Keinan et al. 2007).

### Phylogenetic Analyses

Using a custom python script and SeqKit (Shen et al. 2016), the filtered SNP data sets including variant positions with depth of 5–100 and the outgroup sample were converted into a concatenated multiple sequence alignment in fasta format. For the chloroplast and mitochondrial SNP data sets, RAxML v8.2.11 (Stamatakis 2014) was used to perform 100 rapid bootstrap inferences and ten subsequent maximum-likelihood (ML) searches using a GTRGAMMA model of nucleotide substitution. For the nuclear markers, which were located primarily in genic regions (76.2% of 455.7 K SNPs), RAxML was used with 200 rapid bootstrap inferences and 20 ML searches under the same model.

### Population Genomic Analyses

We used ANGSD v0.931 (Korneliussen et al. 2014) to calculate *F*_ST_ and *D*-statistic without calling individual genotypes. First, we performed read quality filtering in ANGSD. Individuals were discarded from a site based on individual filtered read depth (2–100) at that site. One *S. flexuosum* individual admixed with *S. tenellum* was excluded. We used the Empirical Bayes method implemented in ANGSD to calculate *F*_ST_ using a site frequency spectrum (SFS) to take into account genotyping uncertainty (Fumagalli et al. 2013; Korneliussen et al. 2013). To estimate the SFS, we used ANGSD specifying ploidy level with the command-line argument *-isHap 1* to compute genotype likelihoods (GL) using the SAMTools method (Li et al. 2009) without calling individual genotypes. Allele frequencies were calculated based on GLs using biallelic sites, and minor alleles were inferred from GLs using ML approach (Skotte et al. 2013). Sites were filtered based on the sample size (≥⅓ of individuals). Assuming Hardy–Weinberg equilibrium, we further used ANGSD to estimate site allele frequency likelihood (SAF) jointly for all individuals within each species as well as within each population. Using this estimate, we performed optimization using the expectation maximization (EM) algorithm, and polarization to obtain an ML estimate of the SFS for each species (Nielsen et al. 2012) and an ML estimate of the 2 D (pairwise) SFS for each species pair. This SFS was then folded, and weighted *F*_ST_ was calculated for each species pair using an extended version of the method-of-moments estimator (Reynolds et al. 1983) implemented in ANGSD (Fumagalli et al. 2013). We kept scaffolds longer than 1 M bases equal to 70.3% (278.6) of the total length of the reference.

To compare the estimates based on GLs and on SNP data, the filtered SNP data set was imported into the R statistical environment v3.6.3 (R Core Team 2020) using the package “vcfR” (Knaus and Grünwald 2017) and converted into a genlight object with the package “adegenet” (Jombart and Ahmed 2011). The genlight object was imported into the package “hierfstat” (Goudet 2005) using the package “radiator” (Gosselin 2019), and *F*_ST_ (Weir and Cockerham 1984) was calculated for each species pair. The *F*_ST_ estimates were highly correlated with those calculated in ANGSD for each pairwise comparison (*r*_s_ = 0.81, *P* < 0.0001, supplementary fig. S4, Supplementary Material online), and we hereafter used the estimates inferred with ANGSD.

### *D*-Statistic

We used the multiple-sample version of Patterson’s *D*-statistic (Green et al. 2010) implemented in ANGSD (Soraggi et al. 2018) to calculate genome-wide estimates of introgression. The method is described in detail in supplementary SMM4, Supplementary Material online. Significant deviation of *D*-statistic from zero rejects the null hypothesis about absence of gene flow (Green et al. 2010; Martin et al. 2015). First, we performed read quality filtering in ANGSD and discarded sites missing in more than ten individuals. Individuals were discarded from a site based on individual filtered read depth (2–100) at that site. We kept the scaffolds longer than 1 M bases that equal to 70.3% (278.6 M bases) of the total length of the reference genome. Next, we performed Abbababa2 analysis sampling all bases at biallelic sites in each individual for every triplet of 12 species consistent with the nuclear phylogeny using *Flatbergium sericeum* (Müll. Hal.) A.J. Shaw as the outgroup. The significance of *D*-statistic was accessed by performing Weighted Block Jack-knife method (Busing et al. 1999) using large (1 Mb) blocks to ensure that there is no linkage disequilibrium between the blocks, and that the number of sites within the blocks is big enough to allow the *D*-statistic to be approximated by a normal distribution (Soraggi et al. 2018). Following (Barlow et al. 2018), we chose 1-Mb windows instead of commonly used 5-Mb windows to include scaffolds shorter than 5 Mb into the analysis. The reliability of this approach is confirmed by strong and statistically significant correlation between the *D*-statistic obtained using 5 Mb and using 1-Mb windows (*r*_s_ = 0.85, *P* < 0.0001, supplementary fig. S7*E*, Supplementary Material online). We used a threshold of |*Z*|>3 to reject the null hypothesis which corresponds to *P* < 0.002. The triplets included various triplet combinations of the same species meaning multiple *D*-statistic values were obtained for the same species pairs. Thus, we calculated mean *D*-statistic for species pairs using P_2_ and P_3_ as a pair if *D* for the triplet was significant and positive, and P_3_ and P_1_ as a pair if *D* for the triplet was significant and negative.

### Coalescent-Based Analysis

We reconstructed the coalescent-based phylogeny of the studied species. First, we used ANGSD to generate nuclear genome consensus sequences from aligned reads for each sample. For this, we performed read quality filtering in ANGSD and discarded sites based on filtered read depth (5–100) at that site keeping the scaffolds longer than 1 Mb. The filtered reads were used to generate fasta files for each sample keeping the base with the highest effective base depth (EBD) at each position as implemented in ANGSD. EBD is a product of mapping quality and base quality scores for each base, and it enables more precise base calling for low-coverage sequencing data (Wang et al. 2013). Using the gffread utility (https://ccb.jhu.edu/software/stringtie/gff.shtml#gffread, last accessed March 1, 2021), we extracted spliced genic sequences (CDS) for each sample and used a custom bash script to convert the sequences into multiple sequence alignment fasta files for each gene. For big data sets, the robustness of species tree reconstruction under the coalescent model is not affected by the high degree of missing data when more than one sample per species is sampled (Hovmöller et al. 2013). We kept the first coding sequence in each gene sequence, and filtered the sequences based on the number of missing bases (<50%) and length (>150 bases), and then randomly sampled 1,000 alignments. There were only 1,366 sites in the concatenated nuclear SNP data set that overlapped with the sites located within the resulting gene alignments (0.3% of the SNP data set, 0.08% and 1.2% of the total number of distinct sites and parsimony-informative sites within the gene alignments, respectively). For each of these genes, we used IQ-TREE v1.6.12 (Nguyen et al. 2015) to determine the best substitution model (Kalyaanamoorthy et al. 2017), estimate the best ML tree, and perform 1,000 ultrafast bootstraps (Hoang et al. 2018). We used a coalescent-based phylogenetic method to infer the species tree from a set of the successfully inferred ML gene trees (988 trees) implemented in ASTRAL (Mirarab et al. 2014). ASTRAL is based on the multispecies coalescent model and uses a set of unrooted gene trees, taking a quartet of leaves at a time, to estimate the species tree (Mirarab et al. 2014). We conducted the analysis of the best supported ML gene trees in the multi-individual version of ASTRAL v5.7.3 (Rabiee et al. 2019) to estimate a species tree annotated with posterior probabilities as nodes support. We also estimated a species tree for each bootstrap replicate of each gene tree, and used these species trees to estimate a consensus species tree annotated with node support based on the bootstrap trees. We calculated the concordance factor, that is, the percentage of gene trees recovering the nodes in the species tree, using IQ-TREE v2.0-rc1 (Minh et al. 2020).

### Sliding Window Analyses

To explore the spatial distribution of incongruence across the genome, we generated phylogenetic trees in sliding windows. We generated fasta alignments in the same manner as at the previous step for our coalescent-based analysis, but based on filtered read depth of 2–100, and extracted sequences for 100-kb nonoverlapping windows for each sample using 49 scaffolds longer than 2 M bases that equal to 44% (175.6 M bases) of the total length of the reference. We then used a custom bash script to convert the sequences into the multiple sequence alignment fasta files for each sliding window. We discarded one sample based on the high number of missing bases. We reconstructed the best ML tree for each window in IQ-TREE v1.6.12 (Nguyen et al. 2015) using GTRGAMMA model allowing for a proportion of invariable sites with 1,000 ultrafast bootstrap replicates (Hoang et al. 2018). Using the resulting sliding window trees and the species tree, we calculated the concordance factor in IQ-TREE v2.0-rc1 (Minh et al. 2020) to infer the number of sliding window trees recovering the nodes in the species tree. To estimate how many sliding window trees recovered the species tree topology, we compared the inferred sliding window consensus tree topologies to the species tree topology using the script “findCommonTrees.py” from Edelman et al. (2019). Given that the monophyly was strongly supported for all species in all analyses, we randomly selected and kept one sample per species in all trees for this analysis using the package “ape” (Paradis and Schliep 2019, p. 2019) in the R statistical environment v3.6.3. We used Dendroscope 3 (Huson and Scornavacca 2012), FigTree v1.4.4 (Rambaut 2018), and the packages “ape,” “dendextend” (Galili 2015), “phangorn” (Schliep 2011, p. 2011), and “phytools” (Revell 2012) in the R statistical environment v3.6.3 (R Core Team 2020) to visualize and manipulate the results of the sliding window, coalescent-based, and phylogenetic analyses.

### QuIBL

We made use of QuIBL, a new tree-based method (Edelman et al. 2019), to differentiate between the models with ILS+introgression and with ILS only, and to obtain localized information on introgression. The method is described in detail in supplementary SMM5, Supplementary Material online. To carry out the QuIBL analysis, we used the fasta alignments we generated for our sliding window analyses and kept one sample per species that had the highest sequencing coverage (supplementary table S1, Supplementary Material online). Because *S. compactum* showed strong genetic structure among the populations, we used two samples from two different populations in this analysis. We used 49 scaffolds longer than 2 Mb that equal to 44% (175.6 M bases) of the total length of the reference. Since QuIBL is sensitive to recombination (Edelman et al. 2019), we extracted small 2-kb windows separated by 20 kb from each sample with Seqkit (Shen et al. 2016) to decrease the probability of sampling a window containing a recombination breakpoint (Edelman et al. 2019). We then discarded all windows that had samples with 100% of missing data and generated sliding window trees for the resulting 3,222 windows in the same manner as for our Sliding window tree analysis. We filtered the inferred ML trees based on the number of parsimony-informative sites (≥10), and used the resulted 3,195 trees as an input for QuIBL (https://github.com/michaelmiyagi/QuIBL, last accessed March 1, 2021). The QuIBL output was analyzed in the R statistical environment v3.6.3 (https://github.com/michaelmiyagi/QuIBL/tree/master/analysis, last accessed March 1, 2021), and we used the species tree topology to assign the outgroup to each triplet. We also calculated the percentage of loci supporting discordant topologies and showing significant evidence for introgression. We used the package “lattice” (Sarkar 2008), “corrplot” (Wei and Simko 2017), and “ggplot2” (Wickham 2016) to visualize the results of this analysis and the *D*-statistic tests.

### *D*_FOIL_ Analysis

To test for ancient introgression among the species, we used the *D*_FOIL_ statistic (Pease and Hahn 2015). This extended version of *D*-statistic allows estimating of gene flow direction, and inference of gene flow between the ancestor of a species pair and extant species, and has been widely used to infer recent and ancient introgression, often in combination with the traditional *D*-statistic (Fontaine et al. 2015; Pease et al. 2016; Árnason et al. 2018; Moodley et al. 2020; Vianna et al. 2020). We generated 100-kb fasta alignments for one sample with the highest sequencing coverage per each species (supplementary table S1, Supplementary Material online) in the same manner as described above under “Sliding window analyses,” except that we included all the scaffolds longer than 1 M bases. The window size of 100 kb has been suggested as being sufficiently large to keep the proportion of false-positives very low (Pease and Hahn 2015; Pease et al. 2016; Vianna et al. 2020). We then converted the fasta alignments into *D*_FOIL_ input files (https://github.com/jbpease/dfoil, last accessed March 1, 2021), and performed the test for all symmetrical five-taxon combinations consistent with our nuclear phylogeny with one ingroup clade older than another and *F. sericeum* as the outgroup. We filtered the windows based on minimum total number of sites (>1,000) and minimum number of site counts for any of the *D*_FOIL_ components (>100) per window. We used a χ^2^ goodness-of-fit test with a cutoff of *P* < 0.001 to determine the significance of the inferred introgression signal (Pease and Hahn 2015; Pease et al. 2016). We then estimated the number of consecutive windows showing significant signal of any introgression for each five-taxon combination in the R statistical environment v3.6.3 (R Core Team 2020).

## Supplementary Material

Supplementary data are available at *Molecular Biology and Evolution* online.

## Supplementary Material

msab063_Supplementary_Data
