# Supplementary material for: Extensive Genome-Wide Phylogenetic Discordance Is Due to Incomplete Lineage Sorting and Not Ongoing Introgression in a Rapidly Radiated Bryophyte Genus
Source: Mol Biol Evol. 2021 Mar 3;38(7):2750–66. doi: 10.1093/molbev/msab063 (PMC8233498; doi:10.1093/molbev/msab063)

Supplementary Materials for

Extensive genome-wide phylogenetic discordance is due to incomplete lineage sorting and not ongoing introgression in a rapidly radiated bryophyte genus

Olena Meleshko*, Michael D. Martin, Thorfinn Sand Korneliussen, Christian Schröck, Paul Lamkowski, Jeremy Schmutz, Adam Healey, Bryan T. Piatkowski, A. Jonathan Shaw, David J. Weston, Kjell Ivar Flatberg, Péter Szövényi*, Kristian Hassel, Hans K. Stenøien

*Corresponding authors: email olena.meleshko@ntnu.no, peter.szoevenyi@uzh.ch.

This PDF file includes:
Supplementary Materials and Methods
References
Figures S1 to S11

Supplementary Materials and Methods

SMM1, DNA extraction. Individual capitula of the dried samples were manually cleaned from visible exogenous contamination under a stereo microscope. In order to minimize contamination with the peatmoss ectomicrobiome and environmental DNA, the capitula were thoroughly washed in distilled water and air-dried. The dried tissue was lysed using the Qiagen Tissue Lyser II (Qiagen) for 120s at 30Hz, and total DNA was extracted from cleaned capitulum tissue following the protocol for dried tissue with RNase of the E.Z.N.A. HP Plant DNA Kit (Omega Bio-tek). DNA concentration was measured with the Qubit 2.0 Fluorometer (Thermo Fisher Scientific), and the length of the extracted DNA fragments was quantified on an agarose gel for a subset of the samples. Extracted DNA was fragmented to a mean length of approximately 400 bp via sonication using the Bioruptor Pico (Diagenode) with the following procedure: 5 or 6 cycles of 15 sec 'on' followed by 90 sec 'off'. The fragmentation was confirmed by visualizing the samples on an agarose gel.

SMM2, Testing the library preparation and sequencing. A subset of 11 samples, one sample for each species (except for *S. riparium*), was selected to perform a test library build and sequencing. Individual whole-genome DNA libraries were prepared using customized adapters (Meyer and Kircher 2010) following the Blunt-End-Single-Tube method by (Carøe et al. 2017). The method was developed for application to degraded DNA, but has been successfully used for modern data (e.g. Ribeiro et al. 2019; Bieker et al.) due to its simplicity and low cost. To attach sample-specific dual-indexing barcodes to the fragments, an indexing PCR was performed in 50 µL reactions with 5 µL of library template using custom indexed primers following the protocol for AmpliTaq Gold polymerase (Applied Biosystems) by (Kircher et al. 2012). The thermocycling profile was 10min at 95°C, n cycles of 30s at 95°C, 1min at 60°C and 45s at 72°C, and a final extension step of 5min at 72°C. To avoid the excess of PCR duplicates in the sequencing data, we determined the optimal number of cycles for each library with RT-PCR prior to indexing PCR. Amplified libraries were purified, and quantification and size estimation were performed with the Qubit 2.0 Fluorometer (Thermo Fisher Scientific) and the Bioanalyzer 2100 (Agilent). The libraries were pooled equimolarly based on the molarity of each library within the size range of 420-580 bp, and the pool was size-selected to a mean size of 450 bp using the BluePippin (Sage Science). The quality of the size-selected pool was checked with the Bioanalyzer 2100 (Agilent).

SMM3, Library preparation and estimation of the endogenous DNA content. The rest of the samples were prepared as described above, except that indexing PCR was carried out following the protocol for Herculase II Fusion polymerase (Agilent) by Dabney and Meyer (2012). Number of amplification cycles varied from 12 to 24 between the libraries based on the RT-PCR results. Negative extraction, library build and PCR controls were included in each run. The test sequencing run revealed DNA contamination of various degrees in the samples. Thus, we performed small-scale sequencing for the rest of the libraries to estimate per-sample endogenous DNA content. The libraries were pooled equimolarly into two pools (containing 156 and 62 samples) that were size-selected and quality-checked as described above and sequenced at the NTNU University Museum (Trondheim, Norway) on an Illumina MiniSeq in 150 bp paired-end format. The raw reads were treated in the same manner as described in "Sequencing data processing" in the main text, and for each sample, the ratio between the number of reads aligned to the reference genome and the total number of reads retained after trimming and filtering was used as the endogenous content estimate. Based on this estimate, 16 pools with 7 to 20 libraries each were prepared, size-selected and quality-checked as described above.

SMM4, *D*-statistics. Based on a phylogenetically correct topology for a triplet as $(((P_1,P_2),P_3),\text{outgroup})$, *D*-statistic compares the number of derived and ancestral sites shared between P_1 and P_3 and between P_2 and P_3: *D*=(nABBA-nBABA)/(nABBA+nBABA) where nABBA is the number of sites for which P_1 has an ancestral allele while P_2 and P_3 share a derived allele, and nBABA is number of sites for which P_1 and P_3 share a derived allele and P_2 has an ancestral allele (Green et al. 2010). Even under a scenario of incomplete lineage sorting, given that the $(((P_1,P_2),P_3),\text{outgroup})$ topology is the true topology, P_3 should share the same number of sites with both P_1 and P_2 if there is no gene flow between P_1 and P_3 or P_2 and P_3, so *D* is equal to 0 (Green et al. 2010; Martin et al. 2015). The null hypothesis about no gene flow between the species is rejected when *D*-statistic significantly deviates from 0 (Green et al. 2010; Martin et al. 2015). *D*-statistic can detect ancient and recent gene flow and is robust to different mutation rates and demography in P_1 and P_2 since their divergence, as well as to the effects of ILS (Green et al. 2010; Durand et al. 2011; Patterson et al. 2012; Martin et al. 2015). Traditional Patterson's *D*-statistic uses information from 1 individual per population. We used Abbababa2 algorithm implemented in ANGSD which extends *D*-statistic to use multiple individuals per species by using a weighted sum of the estimated allele frequencies for each individual in every population without calling genotypes (Soraggi et al. 2018). The method provides more accurate estimates of introgression compared to traditional Patterson's *D*-statistic (Green et al. 2010), and performs the best at low and medium-coverage data (1–10x) with sample size of 10 and more (Soraggi et al. 2018).

SMM5, QuIBL. QuIBL provides estimates of introgression proportion and of the likelihood that a locus falls into the model with introgression or with ILS only. For a triplet of species, topologies discordant to the true species tree should have exponentially distributed internal branch length given there is no gene flow among the species. If gene flow took place, the internal branch length distribution will in addition include an element corresponding to the time between the introgression event between the two species and the speciation event of all three species in the triplet (Edelman et al. 2019). QuIBL first estimates the distribution of internal branch length at each locus for a triplets of species. Using the genome-wide distribution, it then uses the expectation maximization algorithm to estimate which distribution each locus falls into, the parameters of the distribution and the likelihood that this distribution corresponds to the model with ILS and introgression (K=2) or with ILS only (K=1). The model that has the lowest Bayesian Information Criterion value is preferred, and a conservative threshold of ΔBIC (>10) is used as a significance criterion (Edelman et al. 2019).

SMM6, Heterozygous SNPs. In case polymorphic paralogous gene regions are falsely mapped to the same orthologous region in the reference genome, variants called from such regions (if not eliminated during our stringent filtering procedures) could potentially be called as heterozygotes. Since all our samples are known haploids, we performed the SNP call specifying ploidy level of 1, thus no heterozygous SNP was called. Therefore, in order to estimate how many sites were potentially heterozygous, we extracted biallelic SNPs for which more than one allele was present in the reads. Following the method used for sample ploidy determination by Yoshida et. al. (2013), we then examined the distribution of read count ratios, and extracted the SNPs falling into the read count ratio of 0.33-0.67, i.e. having nearly equal numbers of reads carrying either of the alleles. We then calculated the percentage of these SNPs to the total number of non-missing SNPs in the sample. We found that, on average per species, 0.25% to 0.59% (Fig. S11) of the variants (0.43% ±0.18% SD per sample, Table S15) fell into this distribution of the read count ratios. The mean significant *D* value across the triplets in our introgression tests was 8%, which is much higher. We therefore conclude that even if these variants were

indeed representing mismapped paralogous regions, the contribution of the latter to the phylogenetic inference and our tests for introgression would be negligible.

References

Bieker VC, Barreiro FS, Rasmussen JA, Brunier M, Wales N, Martin MD. 2020. Metagenomic analysis of historical herbarium specimens reveals a postmortem microbial community. *Mol. Ecol. Resour.* 00:1– 14.

Carøe C, Gopalakrishnan S, Vinner L, Mak SST, Sinding MHS, Samaniego JA, Wales N, Sicheritz-Pontén T, Gilbert MTP. 2017. Single-tube library preparation for degraded DNA. *Methods Ecol. Evol.* 9:410–419.

Dabney J, Meyer M. 2012. Length and GC-biases during sequencing library amplification: A comparison of various polymerase-buffer systems with ancient and modern DNA sequencing libraries. *BioTechniques* 52:87–94.

Durand EY, Patterson N, Reich D, Slatkin M. 2011. Testing for Ancient Admixture between Closely Related Populations. *Mol. Biol. Evol.* 28:2239–2252.

Edelman NB, Frandsen PB, Miyagi M, Clavijo B, Davey J, Dikow RB, García-Accinelli G, Belleghem SMV, Patterson N, Neafsey DE, et al. 2019. Genomic architecture and introgression shape a butterfly radiation. *Science* 366:594–599.

Green RE, Krause J, Briggs AW, Maricic T, Stenzel U, Kircher M, Patterson N, Li H, Zhai W, Fritz MH-Y, et al. 2010. A Draft Sequence of the Neandertal Genome. *Science* 328:710–722.

Kircher M, Sawyer S, Meyer M. 2012. Double indexing overcomes inaccuracies in multiplex sequencing on the Illumina platform. *Nucleic Acids Res.* 40:e3.

Martin SH, Davey JW, Jiggins CD. 2015. Evaluating the Use of ABBA–BABA Statistics to Locate Introgressed Loci. *Mol. Biol. Evol.* 32:244–257.

Meyer M, Kircher M. 2010. Illumina sequencing library preparation for highly multiplexed target capture and sequencing. *Cold Spring Harb. Protoc.* 2010:pdb.prot5448.

Patterson N, Moorjani P, Luo Y, Mallick S, Rohland N, Zhan Y, Genschoreck T, Webster T, Reich D. 2012. Ancient Admixture in Human History. *Genetics* 192:1065–1093.

Ribeiro ÂM, Puetz L, Pattinson NB, Dalén L, Deng Y, Zhang G, Fonseca RR da, Smit B, Gilbert MTP. 2019. 31° South: The physiology of adaptation to arid conditions in a passerine bird. *Mol. Ecol.* 28:3709–3721.

Soraggi S, Wiuf C, Albrechtsen A. 2018. Powerful Inference with the D-Statistic on Low-Coverage Whole-Genome Data. *G3 Genes Genomes Genet.* 8:551–566.

Yoshida K, Schuenemann VJ, Cano LM, Pais M, Mishra B, Sharma R, Lanz C, Martin FN, Kamoun S, Krause J, et al. 2013. The rise and fall of the *Phytophthora infestans* lineage that triggered the Irish potato famine. *eLife* 2:e00731.

Fig S1. Statistics for nuclear PCA. (A) Variance explained by the principal components and (B) Tracy-Widom statistics for each principal component.

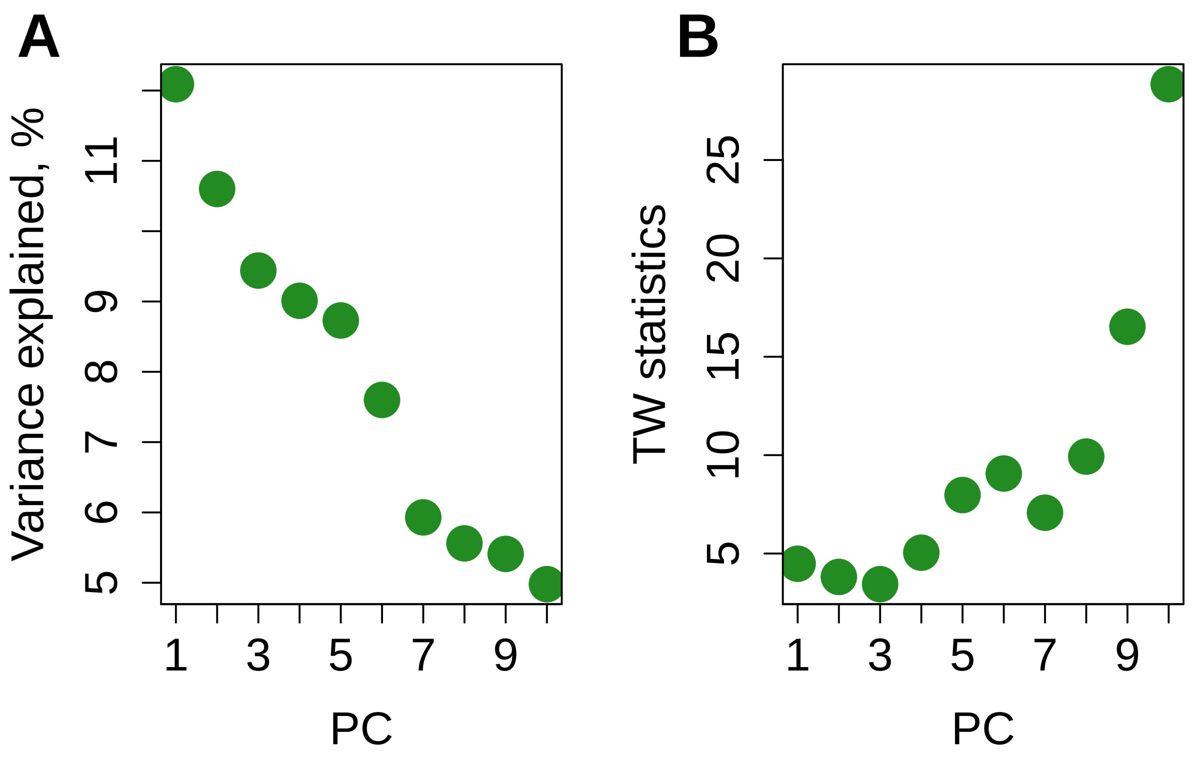

Fig. S2. PCA of *S. capillifolium*, *S. fuscum* and *S. subnitens* individuals (A) PCA of all individuals in the space of the first two principal components and (B) in the space of the first and the third principal components. (C) Variance explained by the principal components. (D) Tracy-Widom statistics for each principal component.

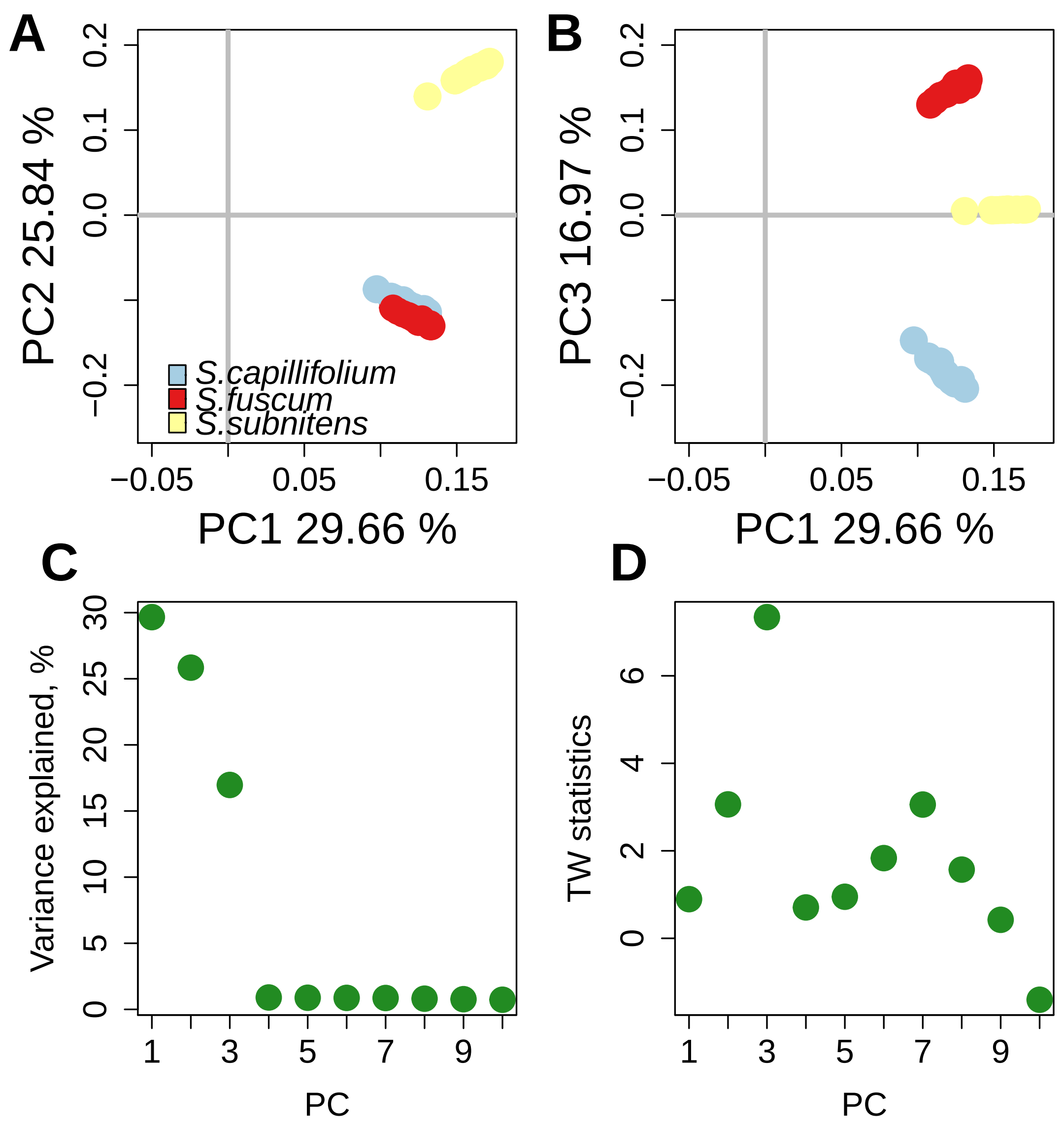

Fig. S3. ADMIXTURE results summary. (A) Boxplot with 10-fold cross-validation error (y axis) in all replicates with the corresponding number of *K* (x axis). (B) Replicates with the lowest 10-fold cross-validation error.

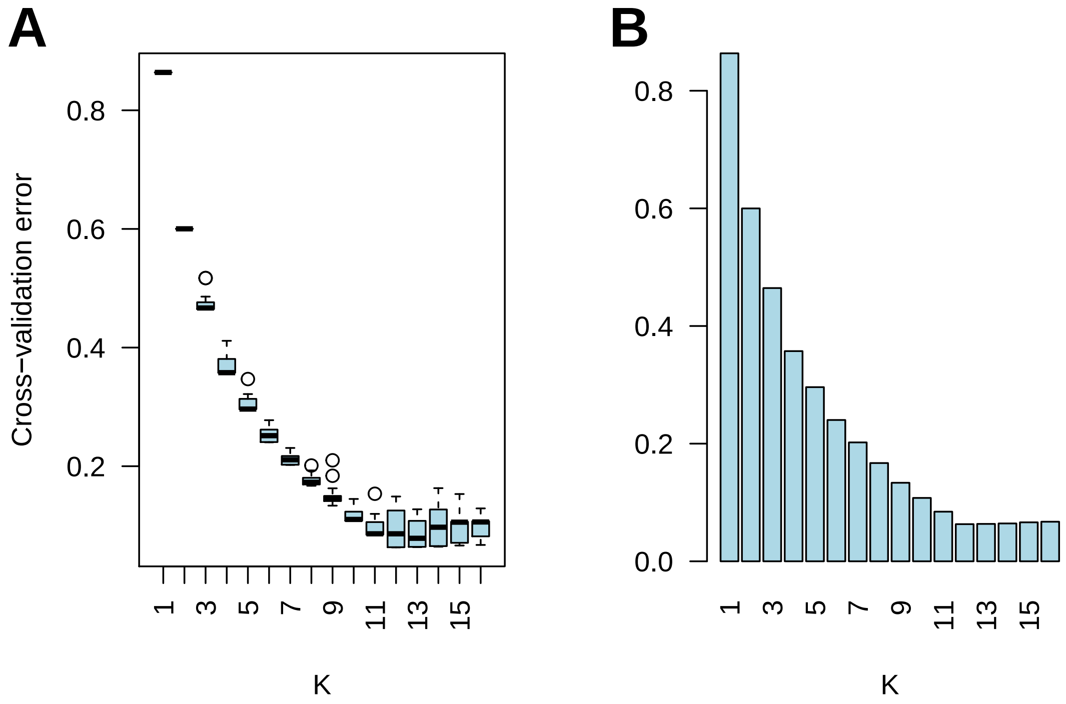

Fig S4. Pairwise F_{ST} among the species (A) based on the SNP data (upper triangle, GATK) and on genotype likelihoods (lower triangle, ANGSD). (B) Relationships between SNP-based and genotype likelihoods based pairwise F_{ST}, the numbers in the right corner correspond to r_S followed by the probability value.

A

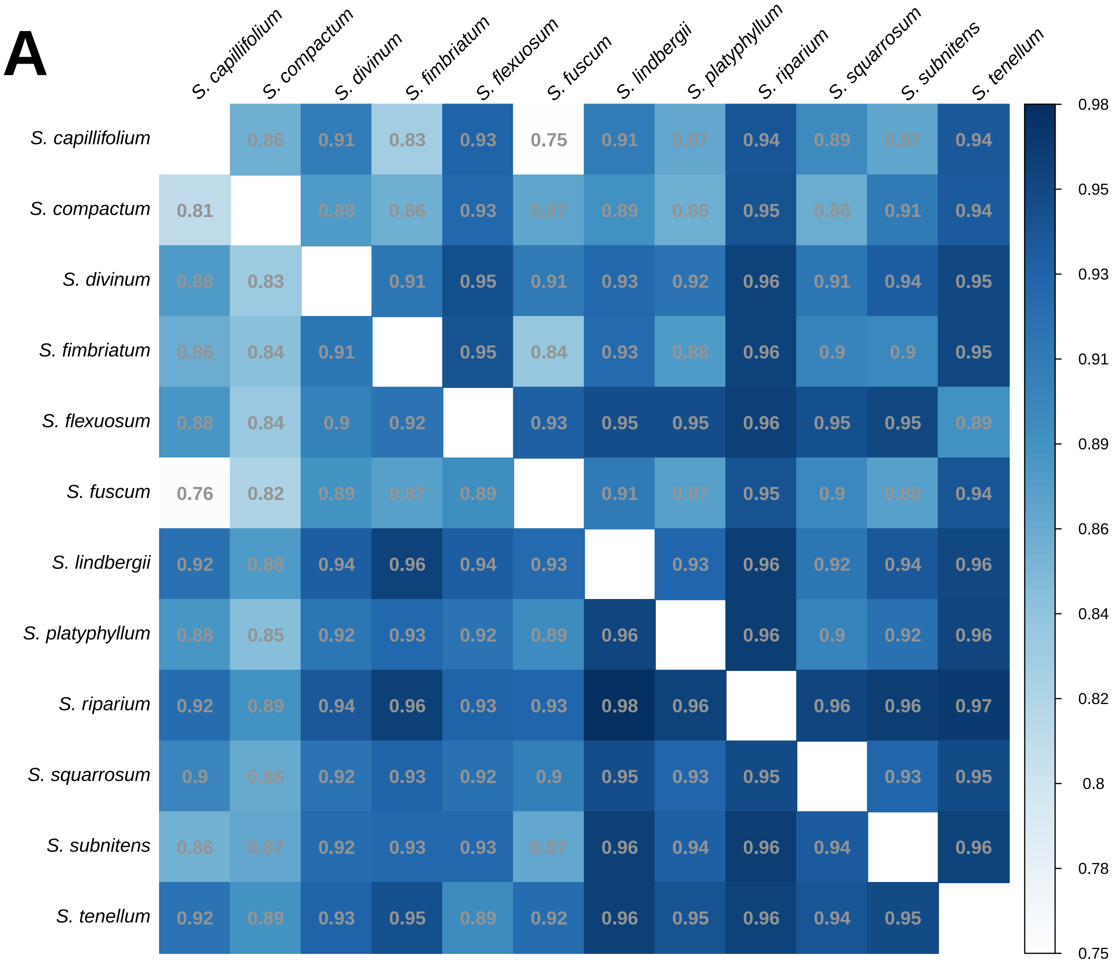

B

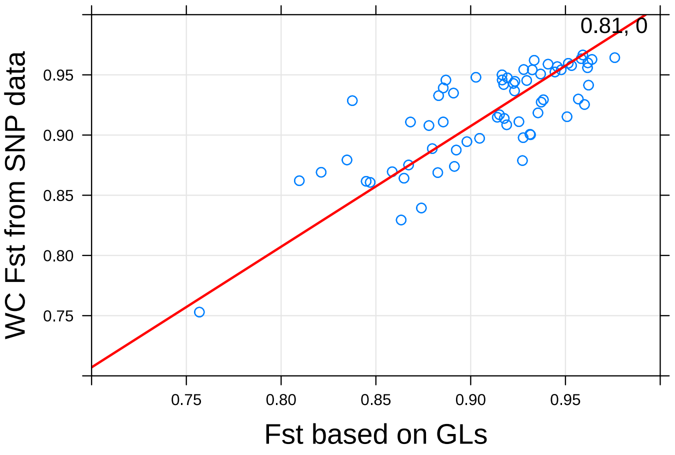

Fig. S5. Phylogenetic relationships among the species inferred using concatenated genetic markers (A) using the nuclear genome, (B) using the chloroplast genome, (C) using the mitochondrial genome. Color of the nodes refers to the bootstrap support of the respective node according to the scale bar shown on the left, all the branch tips within each species were collapsed (triangle symbols). (D) Full nuclear-based tree, color of the nodes and branches refer to the bootstrap support of the respective node according to the scale bar shown on the left.

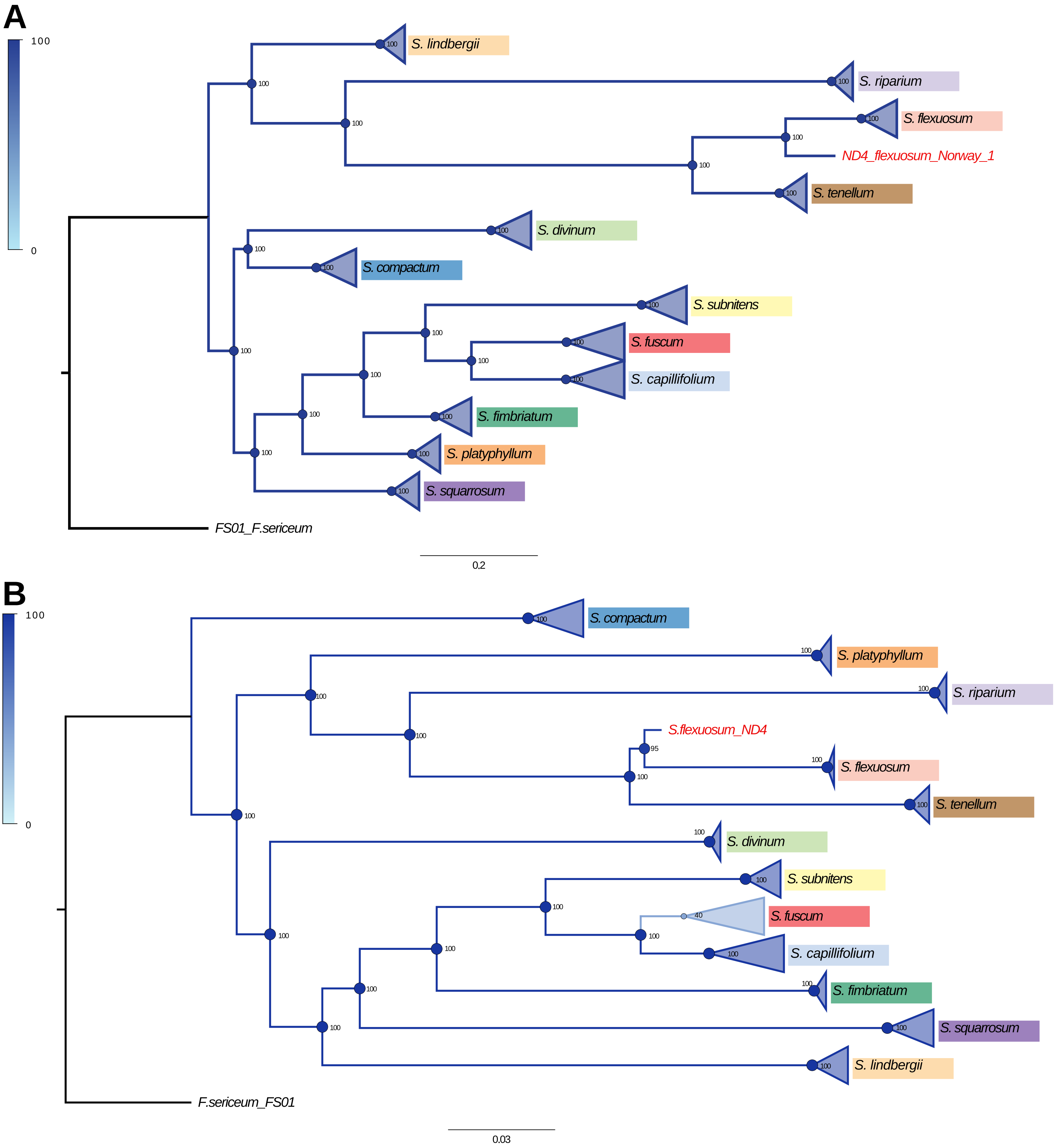

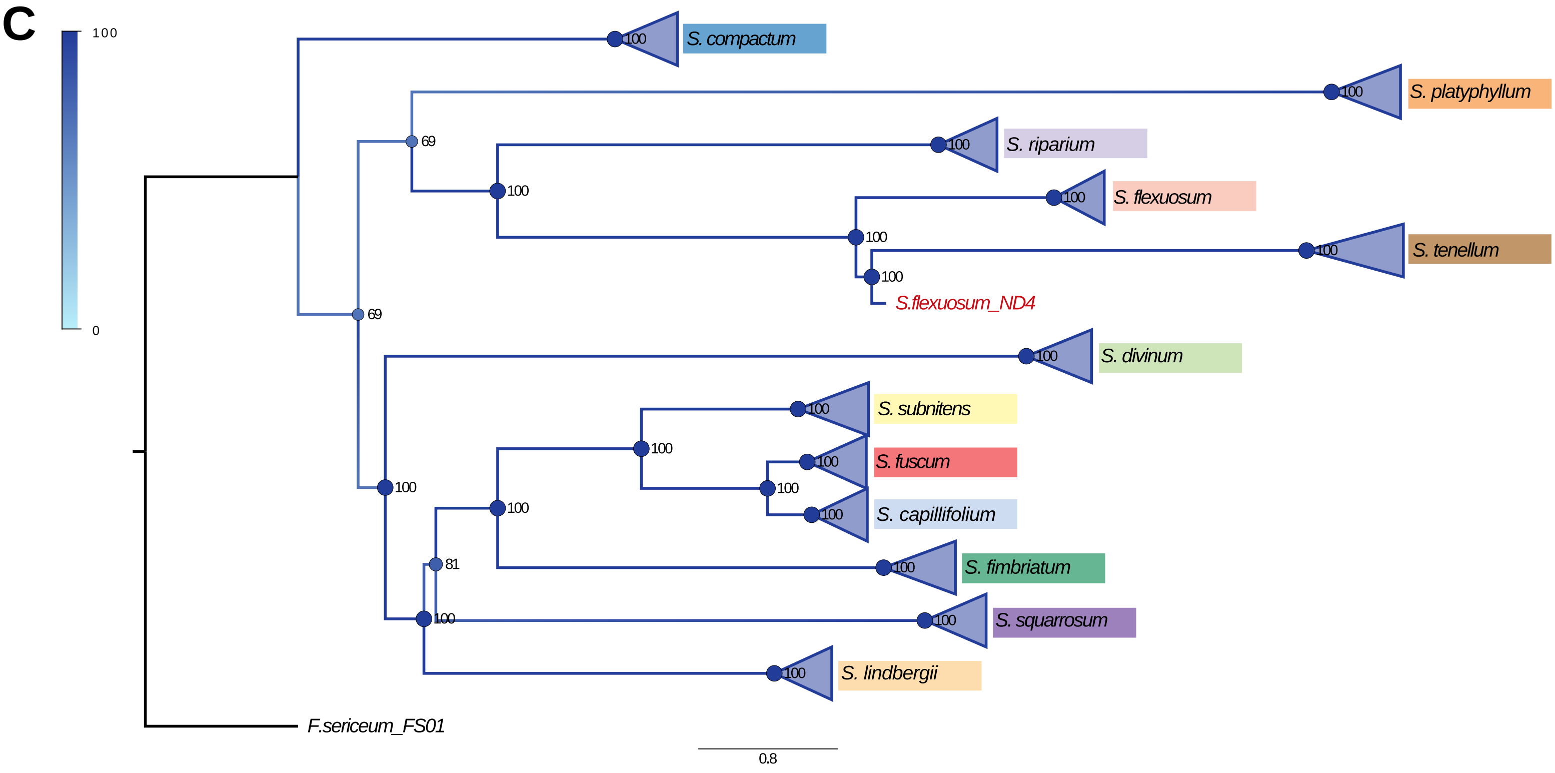
C
100
0
S. compactum
S. platyphyllum
S. riparium
S. flexuosum
S. tenellum
S.flexuosum_ND4
S. divinum
S. subnitens
S. fuscum
S. capillifolium
S. fimbriatum
S. squarrosum
S. lindbergii
F.sericeum_FS01
69
81
0.8

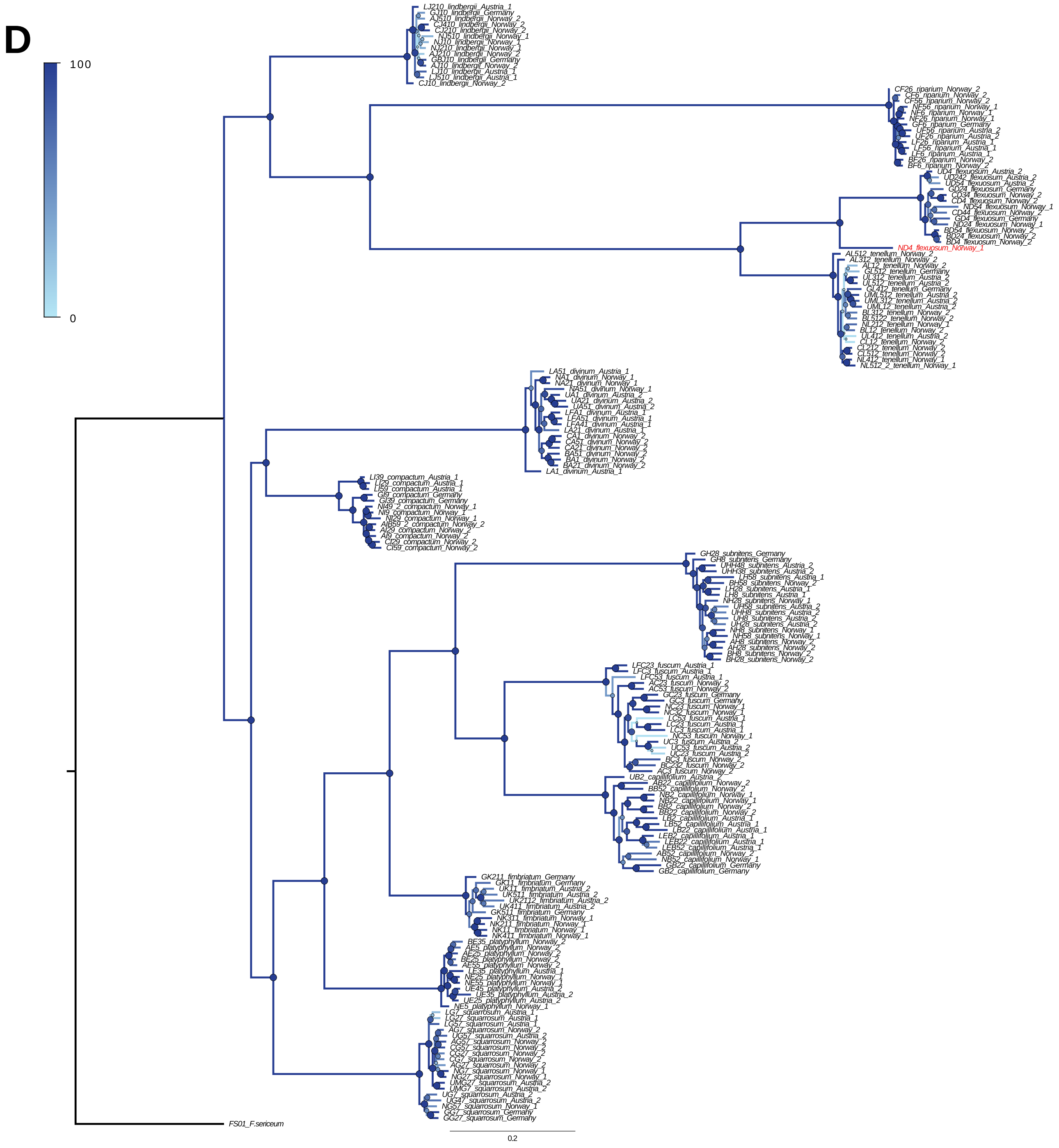
D
100
0
FS01_F.sericeum
0.2

Fig. S6. Coalescent-based and sliding-window analyses (A) Node recovery by the gene trees versus branch length in coalescent units. (B) The consensus tree inferred with sliding window analysis. Color of the nodes refers to the concordance factor as the percentage of sliding window trees recovering the respective node, color of tip branches refers to the species as shown on the right.

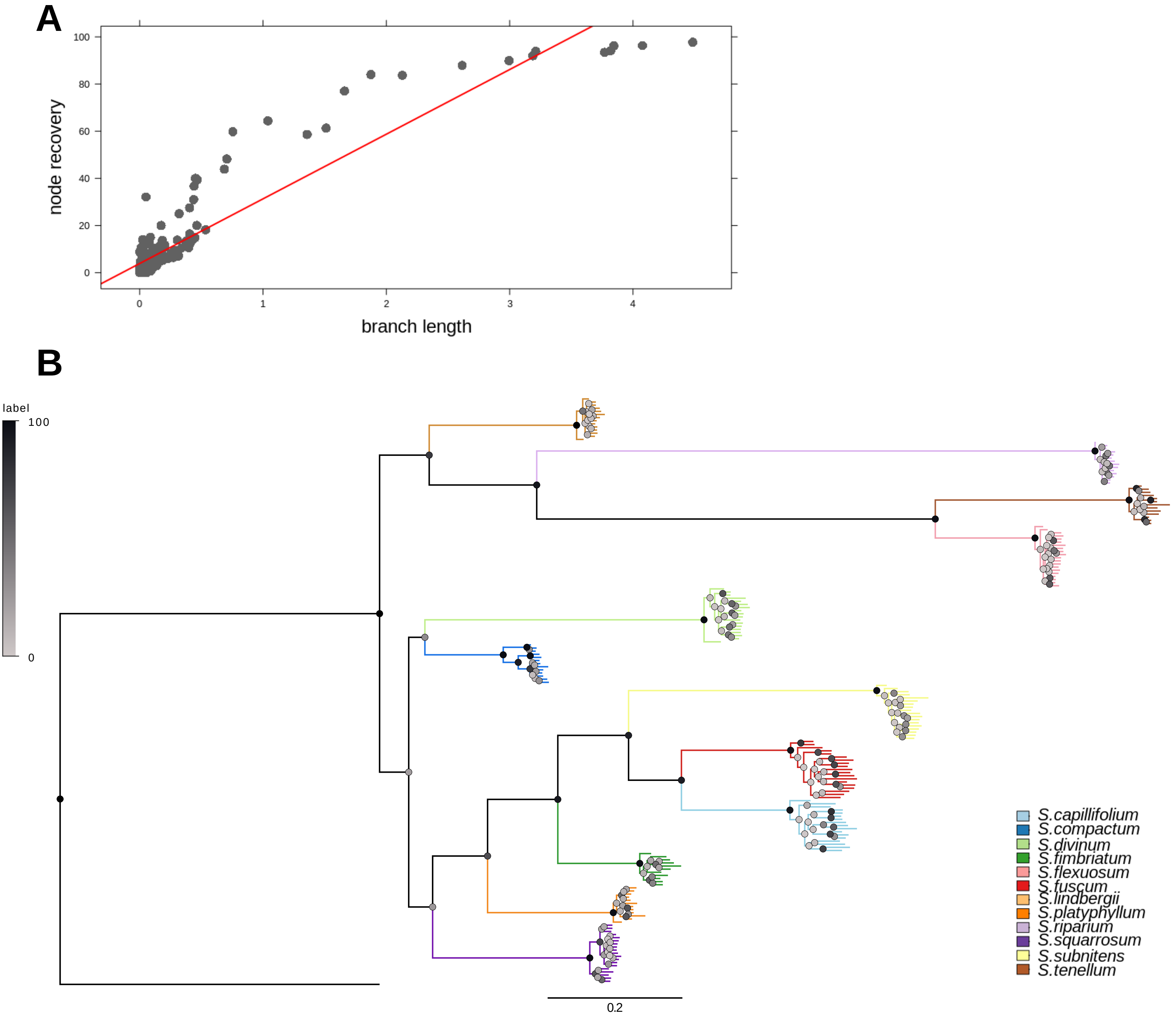

Fig. S7. *D*-statistic using *F. sericeum* as the outgroup in relation to other factors. (A) *D*-statistic, number of sites in all triplets, and number of triplets for a species pair. In all boxplots, the central line is the median, and lower and upper hinges represent the first and third quartiles. (B) The relationships between the number of sites under comparison and absolute *D*-statistic per triplet. (C) The relationships between the number of sites under comparison and average absolute *D*-statistic (calculated using triplets with significant *D* values only) per species pair. (D) The relationship between pairwise absolute average *D*-statistic (calculated using triplets with significant *D* values only) and pairwise F_{ST}. (E) Spearman's rank correlation between *D*-statistic values per triplet obtained with window size of 1-Mbp and 5-Mbp using *F. sericeum* as the outgroup. In all scatterplots, the numbers correspond to r_S followed by the probability value.

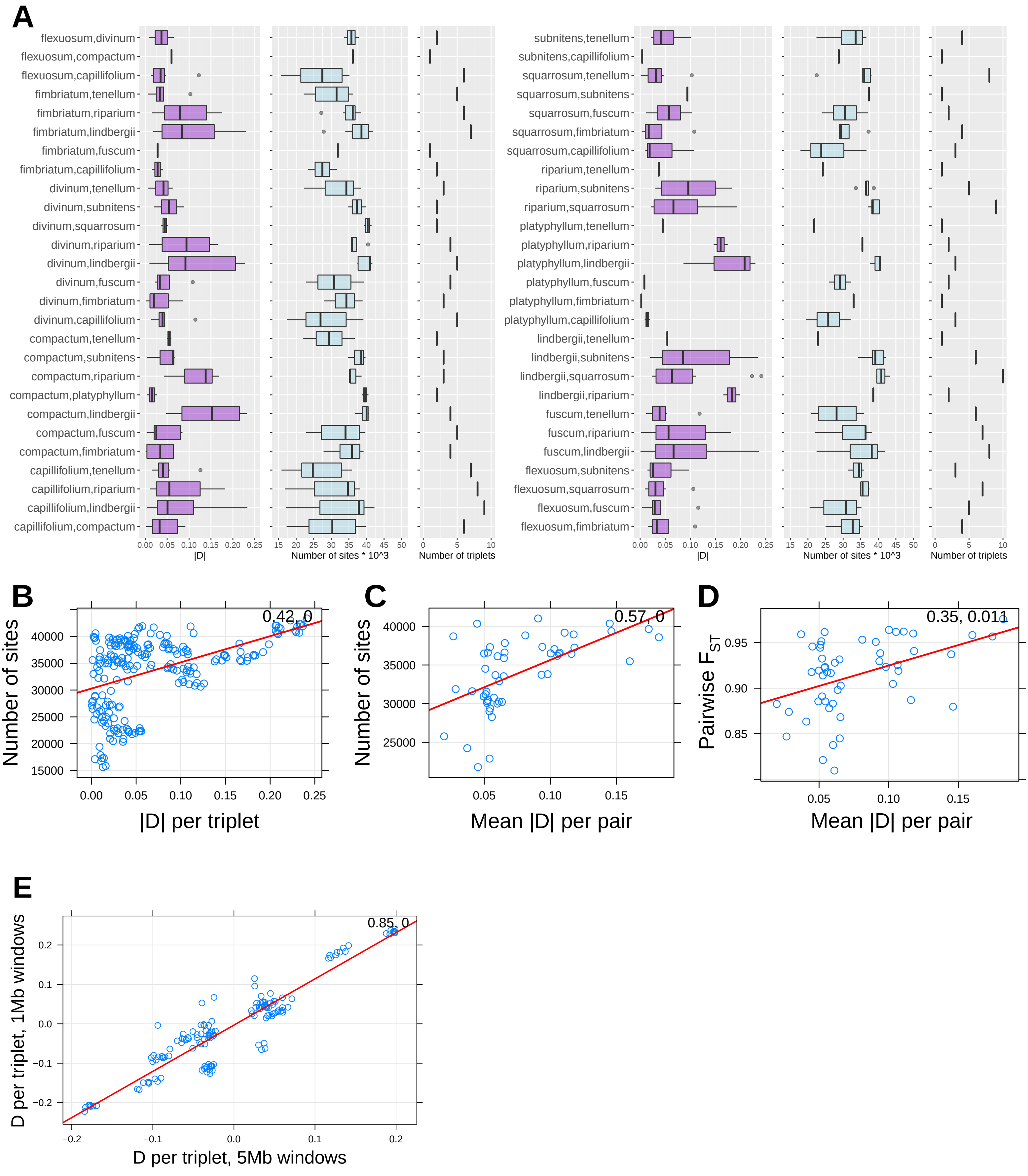

Fig. S8. Migration events reconstructed in TreeMix, the trees inferred with 0 to 5 migration events allowed (top to bottom) are shown on the left. The replicate with the highest likelihood is shown, the numbers correspond to variation explained by the model, standard error and the likelihood of the model. The scale bar on the left represent the migration weight. The residuals of the corresponding model are shown on the right, the scale on the right shows the standard error.

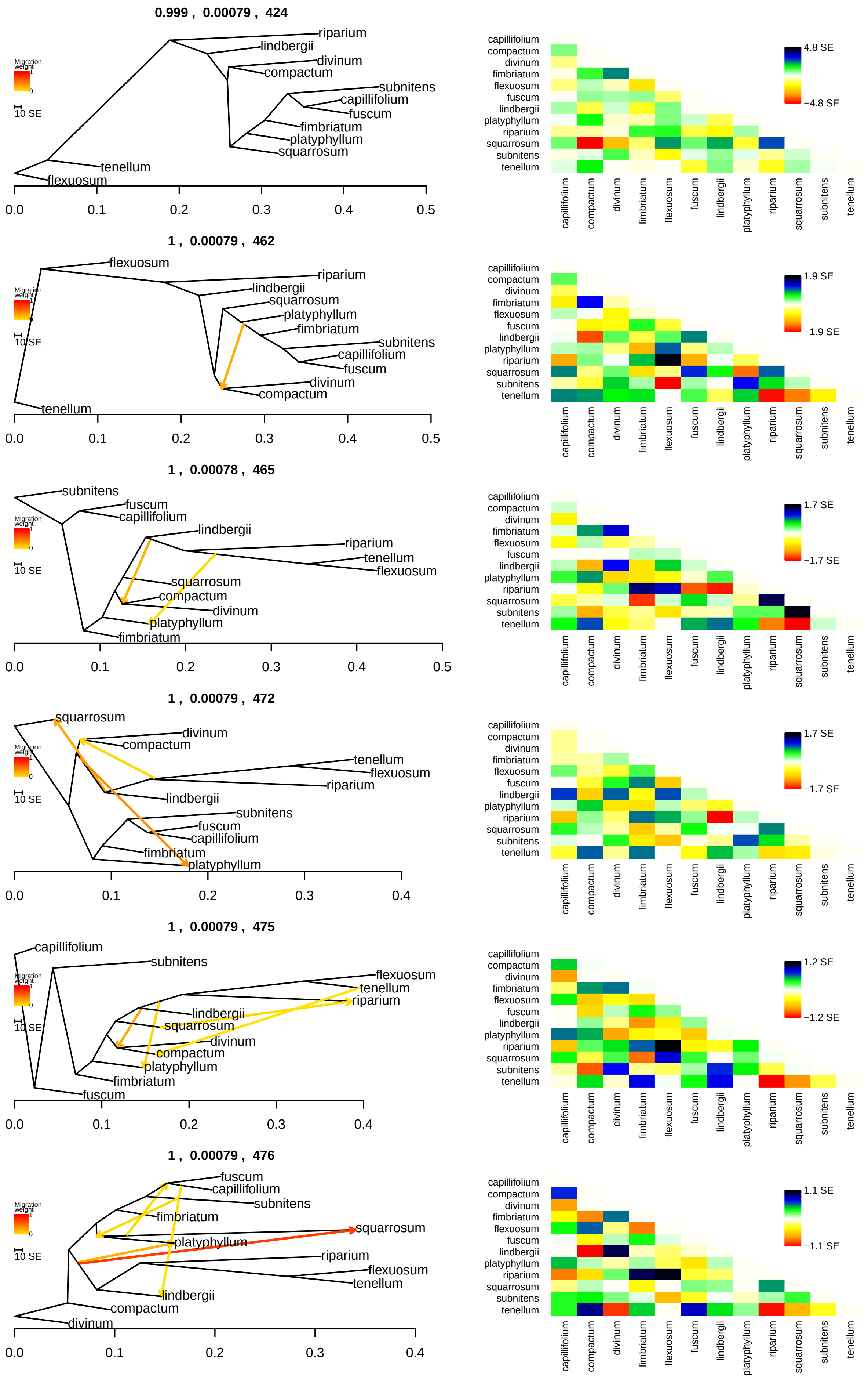

Fig. S9. QuIBL results for the triplets with significant evidence for introgression. (A) Relationships between internal branch length in coalescent units and total proportion of introgressed loci, blue symbols represent triplets with true topology, orange symbols represent triplets with discordant topology. (B) Distribution of average introgression probability for discordant topologies inferred with QuIBL in 2-kb sliding windows across the scaffolds longer than 1 Mb, gray colour corresponds to 20-kb gaps among the sliding windows or to sliding windows with missing data.

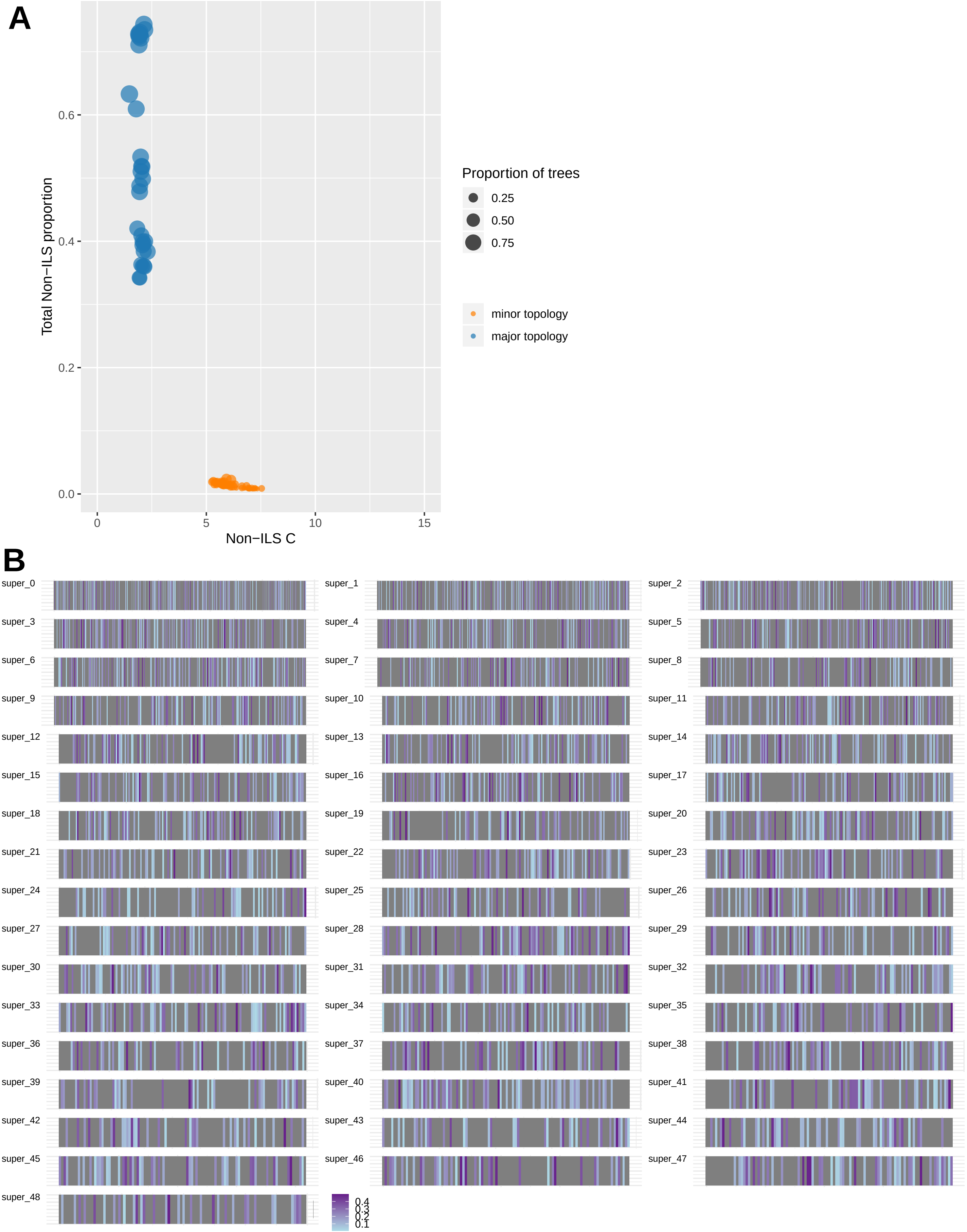

Fig. S10. Number of blocks of consecutive windows showing significant evidence for any introgression among all five-taxon phylogenies. In all boxplots, the central line is the median, and lower and upper hinges represent the first and third quartiles.

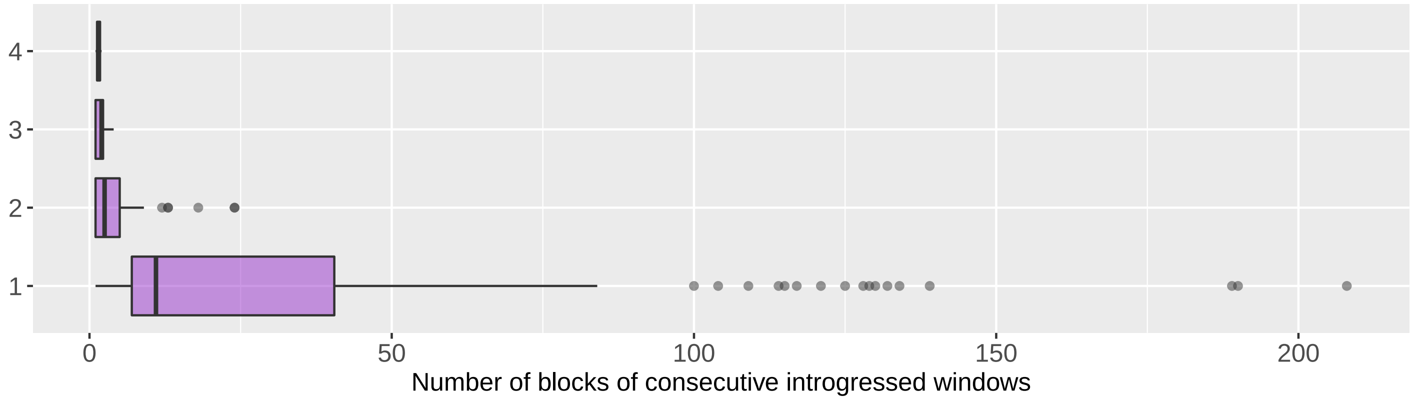

Fig. S11. Percentage of potentially heterozygous variants in all species.

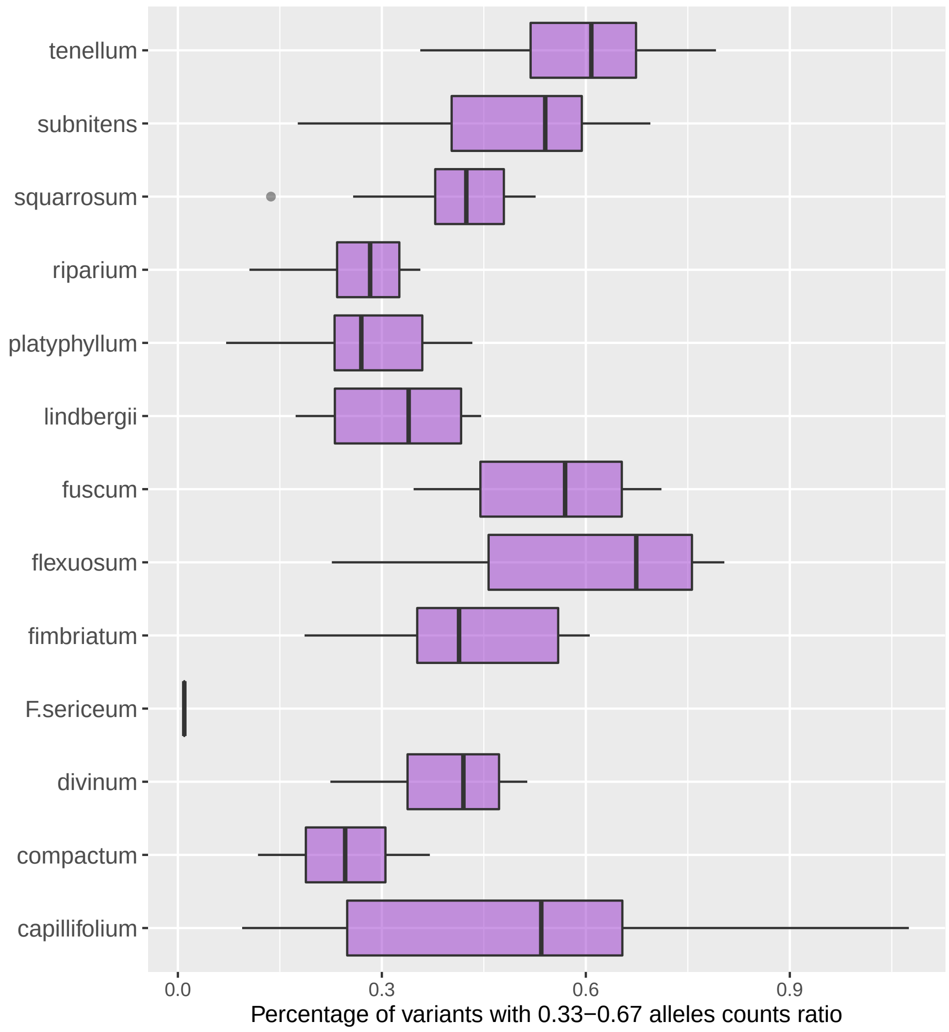

Supplement: msab063_Supplementary_Data [file msab063_supplementary_data.zip › MBE-20-0843.R1_Meleshko_et_al._Supplement_Material_revised.pdf]
